# Mathematical model calibrated to *in vitro* data predicts mechanisms of antiviral action of the influenza defective interfering particle “OP7”

**DOI:** 10.1016/j.isci.2024.109421

**Published:** 2024-03-05

**Authors:** Daniel Rüdiger, Julita Piasecka, Jan Küchler, Carolina Pontes, Tanja Laske, Sascha Y. Kupke, Udo Reichl

**Affiliations:** 1 Department of Bioprocess Engineering, Max Planck Institute for Dynamics of Complex Technical Systems, 39106 Magdeburg, Saxony-Anhalt, Germany; 2 Institute for Computational Systems Biology, University of Hamburg, 20148 Hamburg, Germany; 3 Chair of Bioprocess Engineering, Otto-von-Guericke University, 39106 Magdeburg, Saxony-Anhalt, Germany

**Keywords:** Biological sciences, Biological sciences research methodologies, Natural sciences

## Abstract

Defective interfering particles (DIPs) are regarded as potent broad-spectrum antivirals. We developed a mathematical model that describes intracellular co-infection dynamics of influenza standard virus (STV) and “OP7”, a new type of influenza DIP discovered recently. Based on experimental data from *in vitro* studies to calibrate the model and confirm its predictions, we deduce OP7’s mechanisms of interference, which were yet unknown. Simulations suggest that the “superpromoter” on OP7 genomic viral RNA enhances its replication and results in a depletion of viral proteins. This reduces STV genomic RNA replication, which appears to constitute an antiviral effect. Further, a defective viral protein (M1-OP7) likely causes the deficiency of OP7’s replication. It appears unable to bind to genomic viral RNAs to facilitate their nuclear export, a critical step in the viral life cycle. An improved understanding of OP7’s antiviral mechanism is crucial toward application in humans as a prospective antiviral treatment strategy.

## Highlights

• Systems biology investigation of viral co-infection
• Impact of mutations in OP7 genome on its replication
• Antiviral effect likely mediated by OP7 superpromoter inducing depletion of proteins
• Mechanistic understanding promotes development of OP7 as broad-spectrum antiviral

## Graphical abstract

**Figure:**
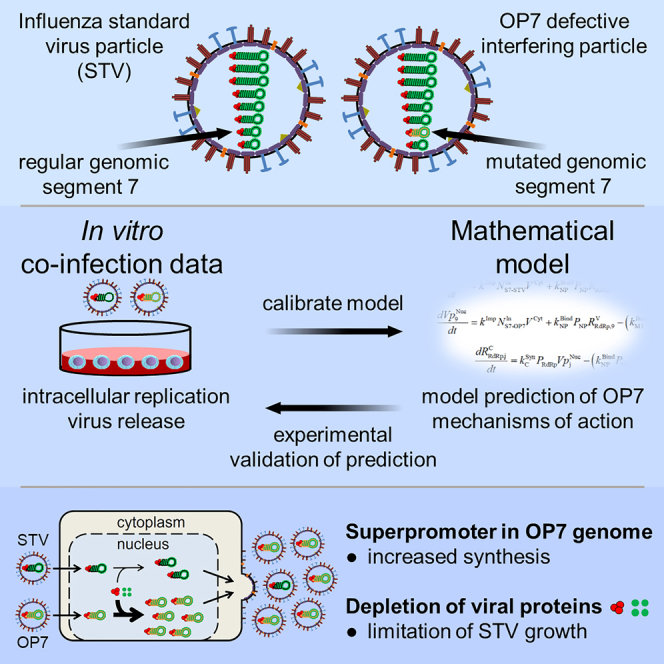

## Introduction

Viral infections cause millions of deaths annually and impose a large socio-economic burden across the world.^1^ While vaccination is the preferred method for prevention of disease, treating acute infections via antiviral therapy is an essential countermeasure to save lives and limit the spread of infections. However, current antivirals might become ineffective due to emerging resistances, e.g., for influenza A virus (IAV).^2^^,^^3^ Thus, the development of broadly acting and readily available antiviral agents is essential for pandemic preparedness. Recently, promising animal studies were performed using antivirals based on virus-derived defective interfering particles (DIPs).^4^^,^^5^^,^^6^^,^^7^^,^^8^^,^^9^

DIPs are structured analogously to their corresponding standard virus (STV), but are rendered replication-incompetent due to mutations in their genome sequence.^10^^,^^11^^,^^12^^,^^13^ For conventional IAV DIPs, this defect is induced by large internal deletions. During co-infection with their STV, DIPs can propagate since the STV provides the missing functions. In this scenario, STV spread is reduced significantly due to the DIP promoting its own replication and competing for viral and cellular resources.^11^^,^^13^^,^^14^^,^^15^ DIPs were found for a large variety of viruses^16^^,^^17^^,^^18^ and their antiviral capability,^19^^,^^20^^,^^21^^,^^22^^,^^23^^,^^24^^,^^25^^,^^26^^,^^27^^,^^28^ mutual competition^29^^,^^30^^,^^31^ and cell culture-based production^4^^,^^5^^,^^32^ have been investigated extensively in recent studies. IAV DIPs inhibit a wide range of IAV strains including human epidemic and pandemic, and even highly pathogenic avian IAV.^8^^,^^33^^,^^34^^,^^35^^,^^36^ Furthermore, DIPs stimulate innate antiviral immunity, i.e., the interferon system.^37^^,^^38^^,^^39^ For IAV-derived DIPs, this enhanced stimulation also enables the suppression of unrelated viruses, e.g., influenza B virus, respiratory syncytial virus, and SARS-CoV2, indicating that they might be applied as broad-spectrum antivirals.^40^^,^^41^^,^^42^^,^^43^

Recently, we discovered a new type of IAV DIP referred to as OP7.^35^ OP7 does not feature internal deletions but contains 37 single-nucleotide substitutions (SNS). It has been shown that OP7 interferes strongly with IAV infection suppressing STV replication more efficiently than conventional DIPs.^4^^,^^5^^,^^42^^,^^43^ The SNS in OP7 occur in the promoter and coding regions as well as in the packaging signal of genome segment 7 (S7). This includes a G3A/C8U substitution that has been shown to induce a “superpromoter” correlated with increased viral genomic RNA (vRNA) synthesis.^44^^,^^45^ In a recent study, mice infected with a lethal dose of influenza STV were rescued by OP7 administration demonstrating its antiviral capabilities.^4^ Currently, the exact mechanism of OP7 interference, the source of its defect in replication and the impact of the SNS on the functionality of its associated proteins, i.e., matrix protein 1 and 2 (M1 and M2), remain elusive.

In the past decades, various mathematical models of IAV infection have been designed to investigate within-host and between-host dynamics.^46^^,^^47^^,^^48^^,^^49^^,^^50^^,^^51^^,^^52^ However, only a few of them focused on the interaction between IAV and DIPs. Previously, we developed models covering different aspects of co-infection, i.e., interference dynamics on the intracellular level,^14^ the population level^53^ and in continuous cultivation systems.^54^^,^^55^ These models almost exclusively considered DIPs with large internal deletions in one of the IAV genome segments encoding for viral RNA-dependent RNA polymerase (RdRp), i.e., segment 1, 2 or 3. In general, they disregarded the synthesis of aberrant viral proteins from the defective interfering (DI) genome, because the large internal deletions would likely render them non-functional. So far, the impact of IAV S7 containing SNS, which might result in the synthesis of a mutated protein affecting virus replication, has not been investigated by a mathematical modeling approach.

To obtain a more detailed understanding of OP7 and STV co-infection, we developed a mathematical model of intracellular virus replication during co-infection. This model was calibrated using experimental data and employed to explore potential effects of the SNS in the genome of OP7. Together, experimental data and model simulations provide a better characterization of the underlying mechanism of OP7 interference in animal cell cultures and support its development as an antiviral agent.

## Results

### OP7 accumulates to high levels and suppresses STV replication in co-infection experiments

To examine how OP7 affects IAV infection dynamics and spreading, we utilized experimental co-infection data from a recent study performed by Kupke et al.^35^ Furthermore, we analyzed the samples generated in this study using an improved assay for real-time reverse transcription qPCR (RT-qPCR) and a newly established mass spectrometry-based protein quantification technique^56^ (see STAR Methods). These methods enabled the differentiation between genome segment 7 of the STV (S7-STV) and of OP7 (S7-OP7) on the RNA levels as well as for the M1 proteins derived from them (M1-STV and M1-OP7).

**Figure 1:**
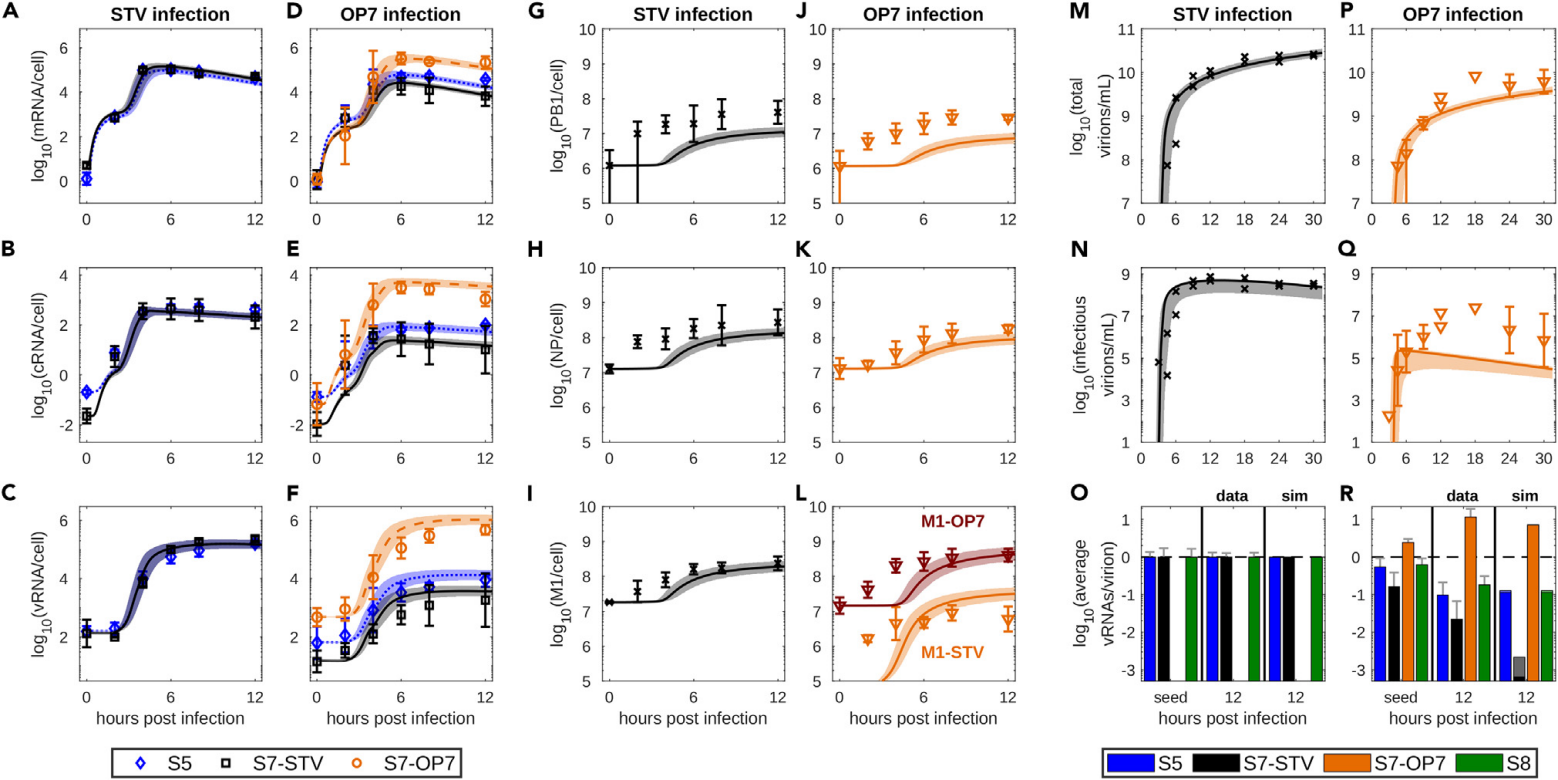
OP7/STV co-infection data and model simulations capturing the virus dynamics Simulations of the final OP7/STV co-infection model fitted to experimental data and 95% prediction bands generated by sampling from the estimated parameter distributions. (A–F) Cell-specific viral RNAs, (G-L) cell-specific viral proteins and (M-N, P-Q) extracellular virus titers for MDCK cells infected with (A–C, G–I, M–O) STV seed (influenza A/PR/8/34, H1N1) or (D–F, J–L, P–R) OP7 seed virus.^35^ Error bars represent the standard deviation of three independent experiments. Individual data points indicate data from a single or two independent experiments. (O and R) Normalized data (see STAR Methods) showing the average vRNA levels of virus particles for the seed virus and after infection compared to model simulations (sim). See also Data S1, Figure S2 and Table S1.

For STV infection, experimental results showed that the m-, c- and vRNA levels of different gene segments follow similar dynamics (Figures 1A–1C). In contrast, during OP7/STV co-infection, the levels of all S7-OP7 RNA species were one to two logs higher than those of S5 RNAs, which were themselves up to one log above the level of all S7-STV RNA species (Figures 1D–1F). S5 mRNA showed similar levels for STV infection and OP7/STV co-infection; yet, S5 cRNA levels were slightly and S5 vRNA levels strongly reduced.

Generally, the protein levels measured during OP7/STV co-infection were reduced, but exhibited dynamics similar to STV infection (Figures 1G–1L). M1-STV showed a decrease by one log during co-infection while M1-OP7 accumulated to significantly higher levels comparatively. Extracellular virus titers presented in Figures 1M, 1N, 1P, and 1Q were taken directly from Kupke et al.^35^ and show that during OP7/STV co-infection the concentrations of total and infectious virions are reduced by one to two logs compared to STV infection.

Lastly, we quantified the average vRNA levels in parental and progeny virus particles. To that end, extracellular vRNA concentrations of S5, S7-STV, S7-OP7 and S8 were measured in the seed virus and after infection at 12 h post infection (hpi). For comparison, the obtained values were normalized (see STAR Methods) to obtain a baseline of a single copy of each vRNA segment per STV particle (Figure 1O). The results of this analysis showed that S7-OP7 vRNA had already accumulated to more than one copy per virion in the OP7 seed virus and that S7-STV was only detected in about 10% of virions (Figure 1R). S5 and S8 vRNA levels were also reduced compared to the STV seed. Over the course of OP7/STV co-infection, these differences were exacerbated as S7-OP7 levels in progeny virions increased even further while the levels of the STV segments were reduced at 12 hpi.

In sum, OP7/STV co-infection led to the predominant accumulation of S7-OP7 RNAs and decreased STV RNA as well as STV protein levels. In addition, the large reduction of virus titers confirms the strong inhibitory capabilities of OP7 against IAV.

### The mathematical model captures STV infection but requires additional changes to describe OP7/STV co-infection

To study the mechanisms of OP7 replication that induce its antiviral effect against IAV infection, we developed a mathematical model of OP7/STV co-infection based on an intracellular model of DIP replication by Laske et al.^14^ Since this model considered a DIP with an internal deletion on genome segment 3, we adjusted it to describe OP7/STV co-infection. To that end, we (1) introduced S7-OP7 as an additional segment in the system, (2) considered M1-STV and M1-OP7 separately and (3) differentiated between viral ribonucleoprotein complexes (vRNPs) bound by M1-STV and M1-OP7 (see Figure S1A and STAR Methods).

Typically, virus infection dynamics differ substantially depending on the cells used and on whether the virus is adapted to the specific cell line.^57^^,^^58^ Therefore, we adjusted relevant kinetic parameters used in the original model. To that end, we calibrated the OP7/STV co-infection model to the STV infection data considering that, in this scenario, no S7-OP7 was present in the virus seed. Thus, we obtained a baseline for kinetic parameters that were fixed for the description of OP7/STV co-infection (Table S1). Following parameter estimation, the model captured STV infection dynamics closely (Figures 1A–1C, 1G–1I, and 1M–1O). While the dynamics of M1 accumulation during STV infection could be described well, the levels of polymerase basic protein 1 (PB1) and nucleoprotein (NP) were slightly underestimated.

Next, we used the baseline parameters to describe the impact of OP7 during co-infection. To that end, we adjusted the viral input to match the values measured in the OP7 seed virus (Figure 1R). Model simulations, however, showed large deviations to the experimental data using this initial model implementation (Figure S2). Therefore, an extension of the OP7/STV co-infection model was required to fully represent the observed infection dynamics.

**Figure 2:**
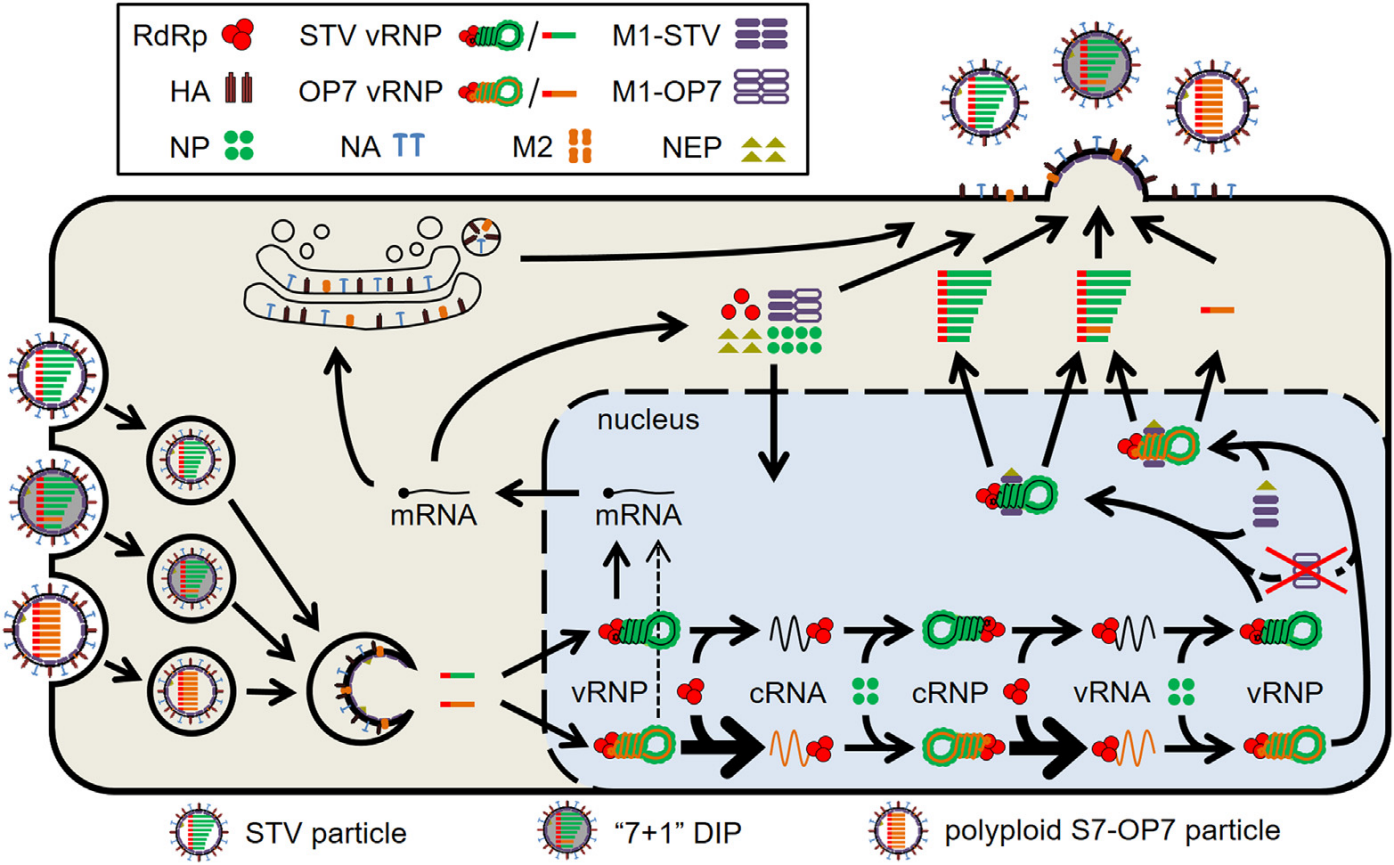
Schematic depiction of the OP7/STV co-infection model Virus entry, nuclear import, viral RNA and protein synthesis, nuclear export, virion assembly and progeny virus particle release during OP7/STV co-infection. The model differentiates between RNA species of individual STV segments and S7-OP7. Different forms of the viral protein M1 can be synthesized from S7-STV or S7-OP7. The model considers a reduced transcription of S7-OP7 mRNA, an increased replication of S7-OP7 cRNA and vRNA, a defect of M1-OP7 affecting the binding of viral genomes in the nucleus, and the existence of three different types of progeny virions (as elaborated in Figures 3, 5, and 6, respectively). Figure adapted from.^53^ See also Figure S1.

The complete co-infection dynamics can be described well when multiple changes to the functionality of S7-OP7 and M1-OP7 induced by the SNS are introduced simultaneously (Figure 2). Considering these changes, the final model captures viral RNA dynamics closely while M1 levels and the infectious virus titer are underestimated (Figure 1). The required changes comprise (1) a replication advantage of S7-OP7 cRNA and vRNA, (2) a reduced transcription of S7-OP7 mRNA, (3) a defect of M1-OP7 affecting the binding of vRNPs in the nucleus, and (4) a disturbed packaging process of S7-OP7 vRNPs (Figure S1B). In the following, we examine these changes individually from the perspective of the final model to show the impact of their inclusion and their effect in comparison to other hypotheses considered.

Taken together, the initial OP7 model was able to capture STV dynamics closely. Yet, to describe OP7/STV co-infection dynamics more precisely, it was required to implement changes regarding the functionality of S7-OP7.

### Model predicts that the SNS found on S7-OP7 vRNA result in a replication advantage and a reduced transcription, confirmed by experimental data

First, we focused on the replication of S7-OP7 vRNA. Previously, two of the specific SNS in the S7-OP7 genome sequence (G3A/C8U) were described to carry a superpromoter, which induces higher cRNA and vRNA synthesis.^44^^,^^45^

**Figure 3:**
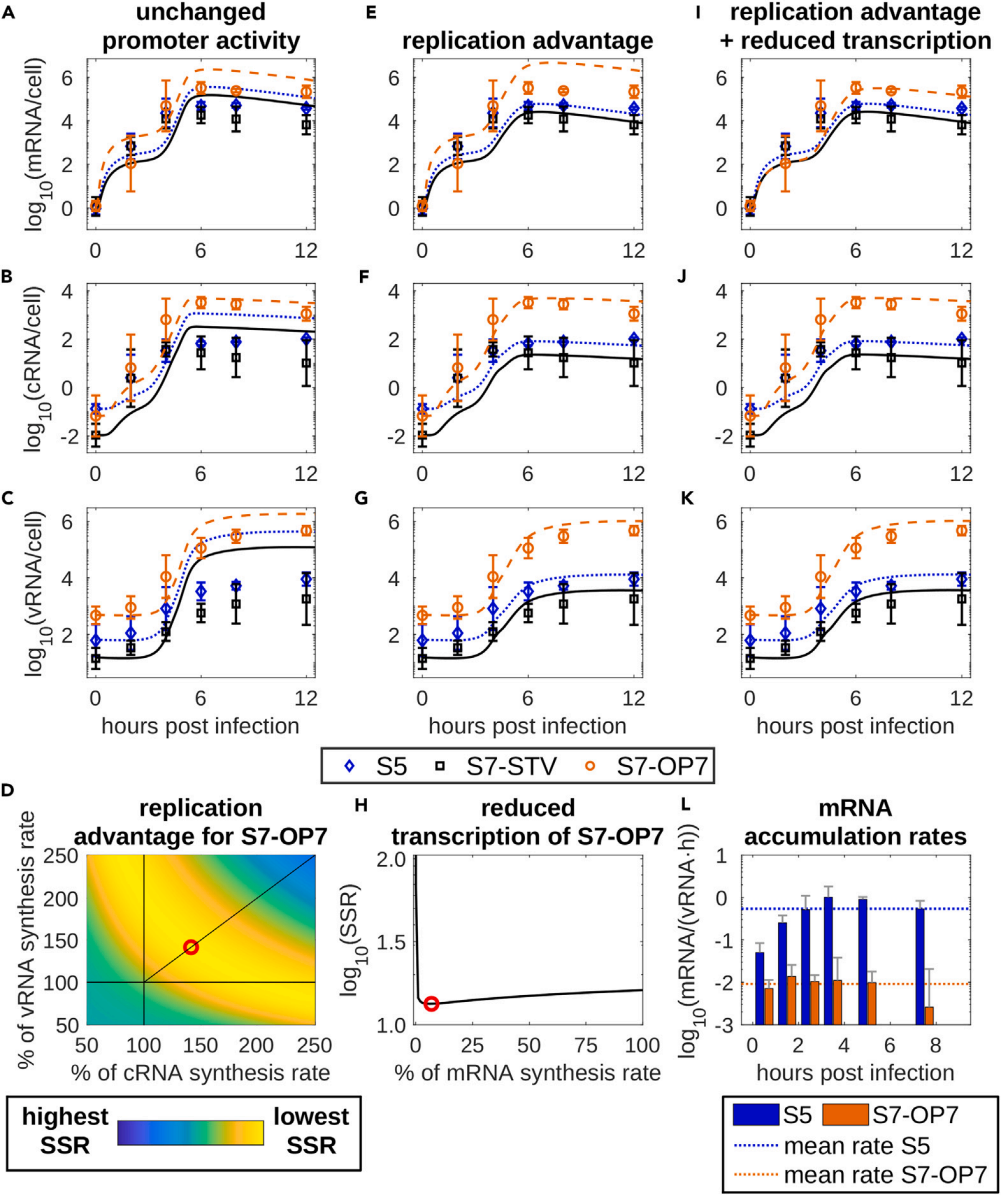
The combination of an increased replication and a reduced transcription of S7-OP7 enabled the description of OP7/STV co-infection dynamics Simulation results of a model describing (A–C) unchanged promoter activity of S7-OP7, (E–G) a replication advantage of S7-OP7 and (I–K) the further addition of a reduced transcription of S7-OP7. For all simulations, it was assumed that M1-OP7 does not bind vRNPs in the nucleus (as elaborated in Figure 5). (D) The sum of squared residuals (SSR) for different combinations of S7-OP7 cRNA and vRNA replication advantages is presented. The red circle represents the best model fit assuming both synthesis reactions are subject to the same advantage. (H) SSR values for the application of reduced S7-OP7 mRNA synthesis rates assuming an existing replication advantage. The red circle represents the best model fit obtained. (L) Experimental data demonstrating differences in mRNA accumulation rates of S5 and S7-OP7 for an OP7/STV co-infection of MDCK cells treated with cycloheximide. Bars depict the calculated mRNA accumulation rates per available vRNA. Error bars represent the standard deviation of three independent experiments. See also Figure S3.

Without changes in promoter activity of S7-OP7 vRNA, model simulations resulted in viral RNA dynamics similar to an STV infection (Figures 3A–3C). The accumulation of the three measured genome segments to distinct levels for all viral RNA species, i.e., S7-OP7 > S5 > S7-STV, can be explained by the initial discrepancy in the viral input (Figure 1R). However, the overall reduction of STV RNA levels and the large deviation between the vRNA levels of S7-OP7 and the other segments present in the experimental data was not captured.

To emphasize the differences between the segments, we incorporated a replication advantage for S7-OP7 in our model. However, it is unclear which step of S7-OP7 replication is improved.^44^^,^^45^ The dynamics of cRNA and vRNA were captured by increasing the synthesis rate of either RNA by 100% or both rates at specific ratios (Figure 3D). We decided to employ the same increase for the synthesis rates of both, cRNA and vRNA, since the superpromoter configuration may affect the promoter regions of both RNAs. Due to the replication advantage, the differences of two to three logs between the vRNAs of S7-OP7 and the other segments could be captured (Figures 3E–3G). Further, the dynamics of S5 and S7-STV mRNA were represented well. However, S7-OP7 mRNA showed elevated levels compared to the experimental data.

To improve the description of S7-OP7 mRNA dynamics, we explored the hypothesis that the SNS on S7-OP7 reduce the transcription of S7-OP7 mRNA. Two previous studies reported contradicting findings regarding the impact of the superpromoter mutation on mRNA levels. Investigating the impact of the superpromoter, Vreede et al. reported substantially inhibited mRNA synthesis^44^ while Belicha-Villanueva et al. observed an enrichment of mRNA compared to STV experiments.^45^ Estimating an optimal rate of S7-OP7 mRNA transcription for the scenario shown in Figures 3E–3G suggested a large reduction (over 90%) compared to the rate of STV mRNA transcription (Figure 3H). The implementation of such a reduction improved the description of S7-OP7 mRNA dynamics (Figure 3I) as indicated by lower values for the Akaike information criterion (AIC, Table S2),^59^ which can be employed to estimate the relative quality of mathematical models regarding specified datasets.

To validate this model prediction, we performed infection experiments using cycloheximide (CHX) and quantified the rate of mRNA accumulation during *in vitro* OP7/STV co-infection directly. CHX prevents the formation of progeny vRNA by inhibiting viral protein synthesis and subsequent replication. Thus, only the parental vRNAs can be utilized as templates for mRNA synthesis, which enables a more accurate determination of mRNA accumulation rates since the bias induced by progeny vRNA synthesis is removed. Based on the CHX experiments (Figure S3), we determined the rate of mRNA accumulation per available vRNA (Figure 3L). Indeed, the experimentally determined mRNA accumulation rates showed that S7-OP7 mRNA was transcribed significantly slower than S5 mRNA, confirming the model prediction.

In sum, model-based analysis predicted that the SNS on S7-OP7 vRNA enhances replication and reduces transcription, which is supported by experimental data.

### The enhanced replication of S7-OP7 vRNA appears to deplete viral proteins and to limit STV vRNA accumulation

**Figure 4:**
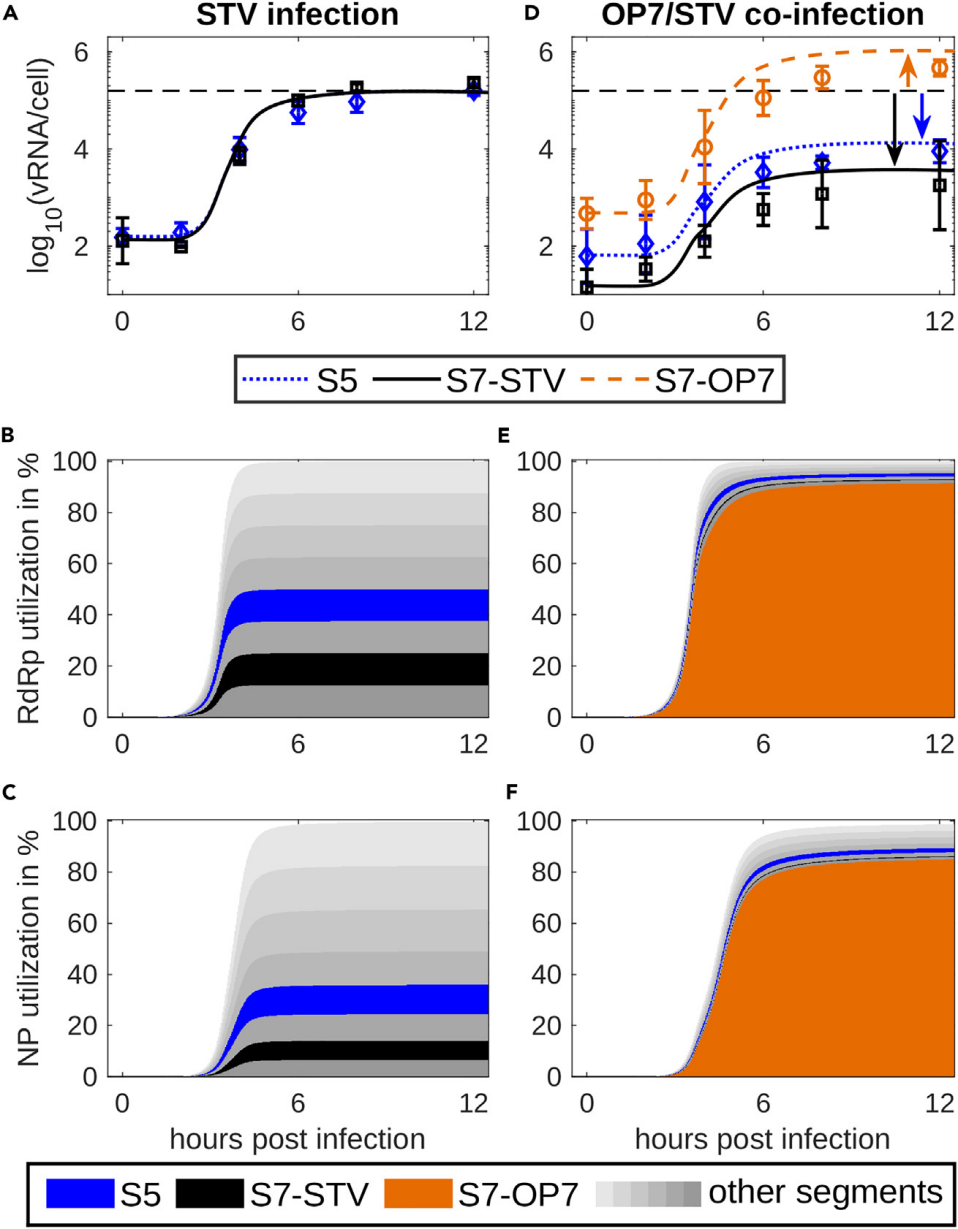
S7-OP7 vRNA replication suppresses the accumulation of STV segments by consuming the majority of viral polymerase and NP Model simulations for (A) an STV infection and (D) an OP7/STV co-infection considering a replication advantage and a defect of M1-OP7 affecting the binding of vRNPs in the nucleus (as shown in Figures 3 and 5, respectively). The black dashed lines represent the maximum vRNA level achieved in an STV infection. Error bars represent the standard deviation of three independent experiments. The stacked bar plots show the simulated utilization of (B, E) polymerase complex RdRp and (C, F) NP for the formation of progeny cRNA and vRNA of the different genome segments for the two scenarios, respectively.

The replication advantage for S7-OP7 introduced in Figure 3 provides an explanation for the high S7-OP7 vRNA levels observed during OP7/STV co-infection. However, the finding that STV vRNA levels were strongly reduced (Figure 4D) suggested a suppression of STV replication by an unknown mechanism. Previous studies discussed the hypothesis that conventional DI vRNAs compete with STV vRNAs for viral resources, e.g., the viral polymerase complex RdRp and NP, during virus replication.^13^^,^^14^^,^^60^^,^^61^ To examine this hypothesis in relation to OP7, we simulated the number of these viral proteins utilized for the formation of vRNAs. Then, we compared these numbers for different segments during an STV infection and an OP7/STV co-infection (Figure 4).

For STV infection, RdRp was equally distributed between the segments (Figure 4B). NP distribution showed a trend toward longer segments, because they require the binding of more NP for stabilization (Figure 4C). For OP7/STV co-infection, the distributions of both proteins were shifted toward S7-OP7, which consumed over 90% of all RdRp and over 80% of all NP available (Figures 4E and 4F). Thus, only low amounts of RdRp and NP could be utilized by other segments reducing their ability to form cRNAs and vRNAs strongly.

Based on these results, we infer that an enhanced S7-OP7 vRNA replication results in the exhaustion of viral proteins required for STV replication, similar to theories postulated for conventional DIPs. Further, we propose that this is the driving factor behind the significant reduction in STV cRNA and vRNA levels, which might, thus, explain the suppressive effect of OP7 on STV infection.

### Model simulations indicate that the deficiency in virus replication of OP7 is induced by a defective M1-OP7 that appears unable to bind vRNPs

**Figure 5:**
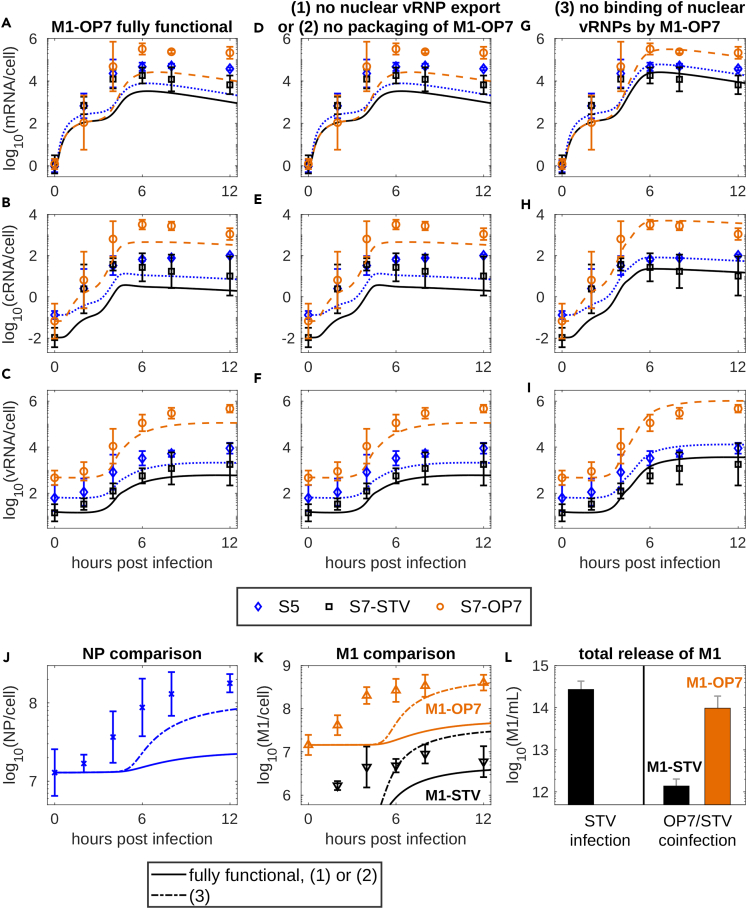
Model simulations suggest that M1-OP7 is defective and cannot bind or only weakly binds vRNPs in the nucleus Simulation results of RNA dynamics for OP7/STV co-infection models describing an M1-OP7 that is (A–C) fully functional, (D–F) cannot be utilized for the assembly of progeny virus particles or prevents nuclear export of bound vRNPs and (G–I) does not bind to vRNPs in the nucleus. (J and K) Comparison of viral protein accumulation in the tested scenarios. For model simulations, it was assumed that S7-OP7 exhibits a replication advantage and a reduced transcription (Figure 3). (L) Experimental data showing concentrations of M1-STV and M1-OP7 in the supernatant at 8 hpi following an STV infection or OP7/STV co-infection. Error bars represent the standard deviation of three independent experiments.

Next, we focused on M1-OP7, which accumulates to over-proportional levels during OP7/STV co-infection, exceeding the level of any other viral protein measured in our experiments (Figures 1G–1L). These enhanced levels could have a strong impact on co-infection dynamics as M1 is an important regulatory protein during virus replication. Initial model simulations of an OP7/STV co-infection considering a fully functional M1-OP7 resulted in the underestimation of all viral RNAs and protein levels (Figures 5A–5C, 5J, and 5K).

Subsequently, we considered that the functionality of M1-OP7 might be affected by the SNS in the S7-OP7 genome sequence. We focused on the main functions of M1, i.e., (1) the nuclear export of vRNPs in conjunction with the nuclear export protein (NEP), (2) its involvement during the packaging of progeny virus particles, and (3) the binding of vRNPs in the nucleus. Disabling the nuclear export of vRNPs by M1-OP7 or its involvement during progeny virion release induced viral RNA and protein dynamics similar to a fully functional M1-OP7 (Figures 5D–5F, 5J, and 5K). To investigate experimentally whether M1-OP7 is indeed packaged and released in virus particles, we analyzed viral protein concentrations in the supernatant at 8 hpi. Following OP7/STV co-infection, the majority of released M1 consisted of M1-OP7 (Figure 5L). This result supports the prediction that M1-OP7 is involved during the packaging and release of virus particles.

Finally, when we considered a defect in the binding of vRNPs in the nucleus, we achieved a close representation of the measured OP7/STV co-infection dynamics (Figures 5G–5K) and the lowest AIC value overall (Table S2). In this scenario, the replication advantage induced to S7-OP7 leads to high S7-OP7 mRNA and M1-OP7 levels. However, if M1-OP7 does not bind or only weakly binds vRNPs to inhibit their replication, they can continue to multiply and achieve the levels observed during OP7/STV co-infection. Without this inhibition, M1-STV levels also increase to higher levels ultimately facilitating the binding and nuclear export of vRNPs. This could also explain why the previous scenarios resulted in overall low RNA levels, as large amounts of an M1-OP7 capable of binding vRNPs would shut down virus replication early. A disrupted binding of M1-OP7 to vRNPs could also provide an explanation on why OP7 is defective in virus replication, typically observed upon OP7 infection without complementation by STV.

Based on the model-based analysis of different hypotheses for the functionality of M1-OP7, we conclude that it likely cannot bind or only weakly binds vRNPs in the nucleus.

### A significant fraction of virus particles produced during OP7/STV co-infection appears to be polyploid with respect to S7-OP7

**Figure 6:**
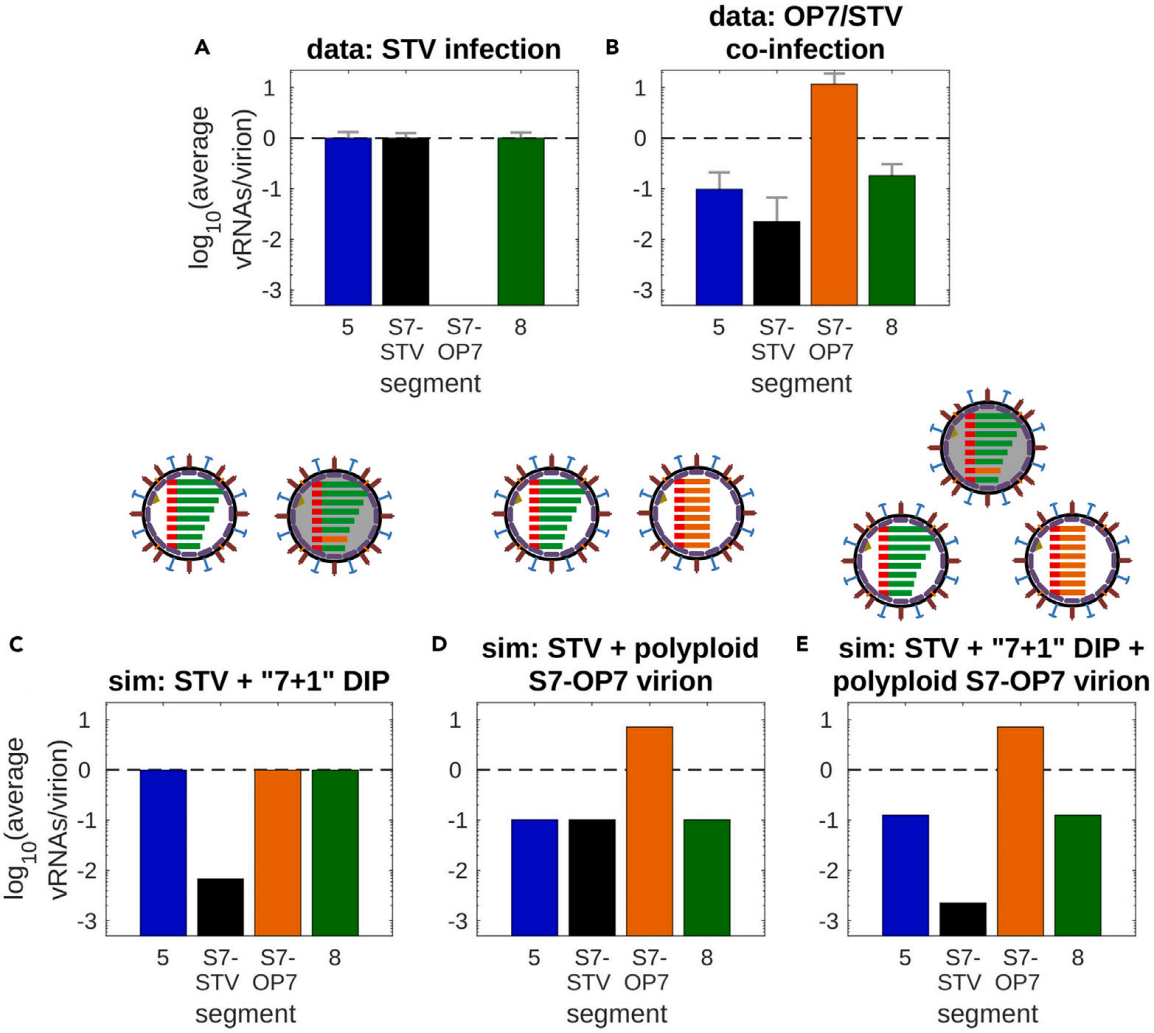
Three different virus particle types are required to describe vRNA levels in progeny virions Normalized experimental data showing the average vRNA levels in progeny virions measured for four different segments at 12 hpi following the infection of MDCK cells with (A) STV seed (influenza A/PR/8/34, H1N1) or (B) OP7 seed virus (see STAR Methods). Error bars represent the standard deviation of three independent experiments. (C–E) Simulations show the average vRNA levels in progeny virions considering the existence of different virus particle types, indicated in the schemes above.

Our experimental data suggested that the average vRNA levels per progeny virion were unequally distributed among the genome segments after an OP7/STV co-infection (Figure 1R). Thus, we tested different hypotheses of virus particle composition that could induce such an imbalance. For STV infection, progeny virions typically contain one vRNA per segment at 12 hpi (Figure 6A). Yet, for OP7/STV co-infection, experimental data showed significantly increased levels for S7-OP7 vRNA (Figure 6B). Moreover, average levels of S5 and S8 vRNAs in virions were decreased by one log and S7-STV was reduced even further. In previous DIP models, it was assumed that two types of virions can be formed, i.e., STV particles containing the eight STV vRNAs (S1-S8) and DIPs containing seven STV vRNAs and a DI vRNA that replaces its corresponding STV segment (“7 + 1” formation).^14^^,^^53^ Implementing this approach in the model resulted in a reduction of average S7-STV vRNA content; yet, it did not result in an increase of S7-OP7 vRNA or a decrease of S5 and S8 vRNAs per virion (Figure 6C).

Since we observed that S7-OP7 accumulated to approximately ten vRNAs per virion at 12 hpi, we next considered the hypothesis that virions containing exclusively S7-OP7 vRNAs are released during OP7/STV co-infection (in addition to STV particles). A similar scenario was already subject of speculation in previous studies.^35^ This approach enabled the description of the increased average S7-OP7 vRNA content and reduced STV vRNA levels in virus particles (Figure 6D). However, a large reduction of S7-STV vRNA levels was not achieved.

Finally, we combined the previous approaches considering the release of three different kinds of progeny virus particles during OP7/STV co-infection, i.e., (1) STV particles, (2) “7 + 1″ DIPs, and (3) OP7 particles that only contain multiple S7-OP7 vRNPs. In this scenario, model simulation could qualitatively capture the experimental data (Figure 6E).

In sum, model simulations indicate that progeny virus particles that are polyploid with respect to S7-OP7 vRNA are formed during OP7/STV co-infection besides STVs. To describe the specific reduction of the average S7-STV vRNA levels in progeny virions, the formation and release of conventional “7 + 1” DIPs is necessary.

### Model predictions describe S7-OP7 vRNA accumulation and STV suppression of serial infection experiments

**Figure 7:**
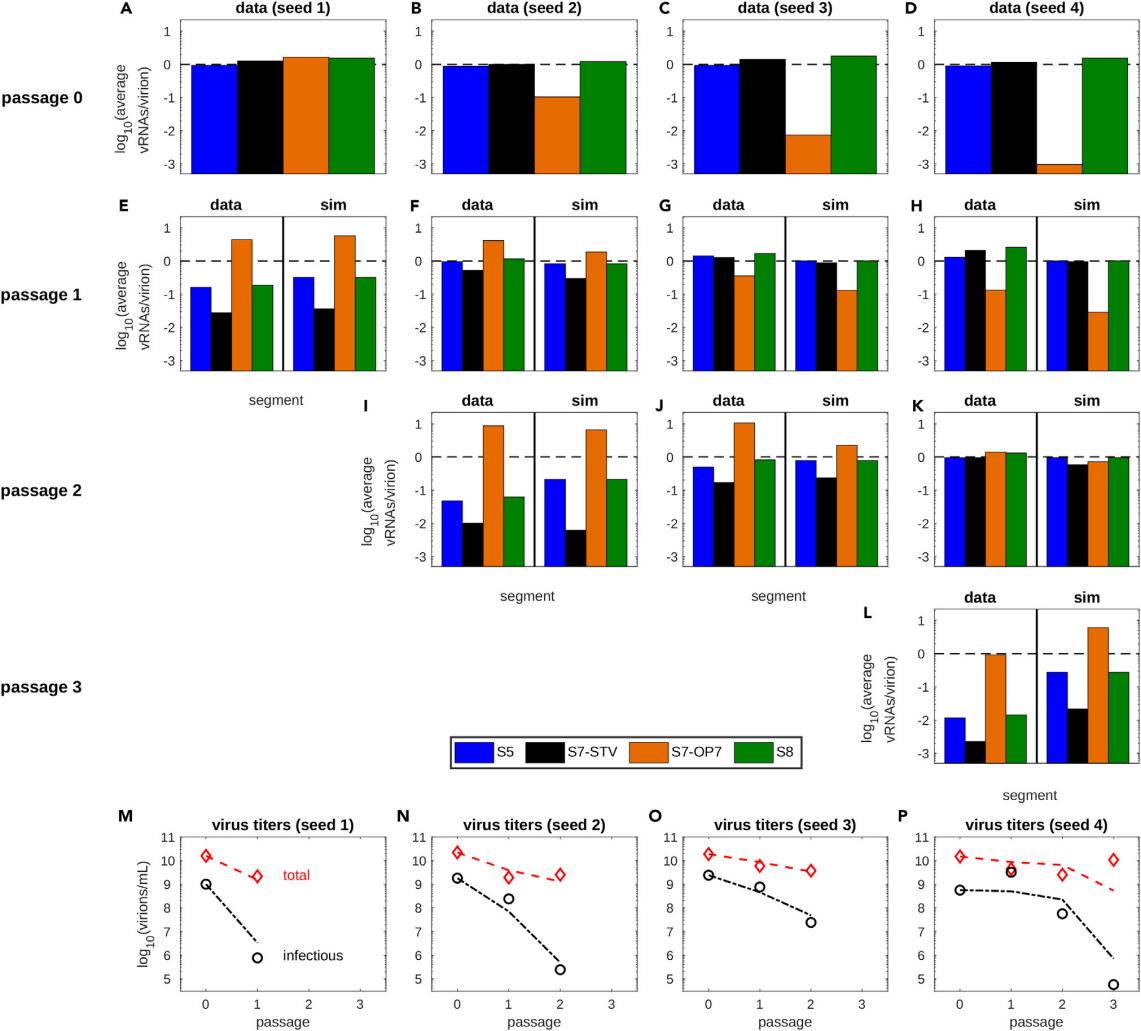
Model predictions describe the accumulation of S7-OP7 vRNA and the suppression of the STV over multiple *in vitro* co-infection passages (A–D) Average vRNAs per virion measured for four segments of different OP7 seed viruses (passage 0). (E–L) Comparison of experimental data and predictions of the final version of the model (sim) following the infection of MDCK cells at 12 hpi after up to three passages. For model simulations, either the original seed virus (passage 0) or the prediction of the previous virus passage was used as an initial condition. Measured vRNA levels were normalized (see STAR Methods). (M–P) Symbols represent experimental data of total and infectious virus particle concentrations determined in the seed virus and after serial OP7/STV co-infection passaging at 12 hpi. Curves depict the corresponding model predictions. See also Figure S4.

Lastly, we tested the predictive power of the OP7 model by challenging it with additional experimental data obtained from serial OP7/STV co-infection experiments. To that end, we started co-infections with seed viruses showing different average S7-OP7 vRNA contents in virus particles (Figures 7A–7D). At 12 hpi, vRNA levels in progeny virions (Figures 7E–7H) and virus titers were quantified. Subsequently, further co-infections were performed using the virus material obtained in these infections (Figures 7I–7L).

For model simulation, we applied the vRNA levels measured in the corresponding seed viruses (Figures 7A–7D) as initial conditions for the first passage. To simulate further passages, the respective vRNA levels predicted at 12 hpi of the corresponding previous passage were used to adjust the initial conditions. We tested the hypotheses of virion release shown in Figures 6C–6E for model prediction to decide which is suited best for the final version of the model. Initially, a release of STV and conventional “7 + 1” DIPs resulted in the worst model fit (Table S2). This was confirmed by the model prediction for multiple passages, which did not induce the accumulation of S7-OP7 vRNAs observed for all tested conditions. Employing a release of STV and OP7 particles, but no conventional “7 + 1” DIPs, did not enable the description of the reduction of S7-STV vRNA observed in the passaging experiments (Figure S4). In contrast, model simulations assuming the release of all three particle types predicted the average vRNA levels in progeny virions well for passage 1–3 for all four initial conditions (Figures 7E–7L). Further, the reduction of virus titers during serial infections was described closely (Figures 7M–7P). The related AIC values also show a clear trend toward the three-particle hypothesis (Table S2), making it preferable for the final OP7/STV co-infection model.

Taken together, the OP7/STV co-infection model predicted the accumulation of S7-OP7 vRNA, the suppression of the STV segments and the reduction of virus titers induced by OP7 in serial infection experiments for up to three passages and varying initial conditions.

## Discussion

In this study, we utilized experimental data to develop a mathematical model of OP7/STV co-infection. The model was employed to investigate the mechanisms of OP7 interference with the STV and the source of its defect in replication, which were yet unknown.

The replication of S7-OP7 vRNA appears to be strongly increased by the SNS in its sequence. In particular, it contains the well-characterized superpromoter substitution (G3A/C8U) that is related to increased cRNA and vRNA synthesis.^44^^,^^45^ For conventional DIPs, a similar replication advantage has been proposed. However, it is based on the significantly shorter length of their viral genome leading to faster replication.^11^^,^^13^^,^^14^^,^^62^ Therefore, OP7 and conventional DIPs both show a similar phenotype, i.e., an enhanced replication of the DI vRNA. But interestingly, this appears to be based on different genotypes. In a previous modeling study, we estimated the replication advantage of a well-characterized conventional DIP “DI244”,^33^^,^^63^ which resulted in a cRNA synthesis rate increased by 32% compared to STV segments.^53^ Applying a similar implementation to our OP7/STV co-infection model, S7-OP7 would be subject to a higher increase in cRNA synthesis, i.e., by 100% (Figure 3D). This result is in line with our observation that OP7 shows a higher interfering efficacy than conventional DIPs *in vitro* and *in vivo*.^4^^,^^5^^,^^42^^,^^43^ Together, our findings support the hypothesis that the replication advantage of OP7 constitutes at least a part of its antiviral effect, as elaborated in the following.

The implementation of a replication advantage for S7-OP7 vRNA did not lead to an increase of its levels, but rather induced a decrease of STV vRNA levels by one to two logs during OP7/STV co-infection (Figures 3A–3G). To identify the underlying mechanisms, we evaluated if a competition for viral proteins could induce such an effect, as previously suggested for conventional DI vRNAs.^13^^,^^60^^,^^61^ Model simulations estimating the number of proteins utilized showed that S7-OP7 consumes a large portion, i.e., more than 80%, of available RdRp and NP (Figures 4E and 4F). Thus, by replicating faster, S7-OP7 cRNA and vRNA likely accumulates early capturing viral proteins before the STV segments generate enough templates to amplify their replication. Therefore, the depletion of available viral proteins by S7-OP7 could provide an explanation for the suppressed replication of STV vRNAs. This could constitute the inhibitory effect of OP7 during STV co-infection. In line with this, model-based analysis of the replication of a conventional DIP with a deletion in segment 3 showed that the competition for viral resources induces a similar suppression of STV replication.^14^ Interestingly, OP7 seems to establish such a DI-like behavior via the superpromoter on S7-OP7 instead of a large internal deletion.

Based on our model simulations, we found clear indications that M1-OP7 is defective and cannot bind (or only weakly binds) to vRNPs in the nucleus (Figure 5). This binding step is crucial for the regulation of IAV infection, since the wild-type M1-STV typically inhibits vRNP replication and subsequently initiates the nuclear export of vRNPs.^64^^,^^65^^,^^66^ If M1-OP7 does not bind to vRNPs, viral genomes are not exported to the cytoplasm for packaging or release. A deficient M1-OP7 could, therefore, explain the defect of OP7 particles in virus propagation, typically observed in our experiments.^4^^,^^35^ Accordingly, OP7 likely requires a supplementation of M1-STV to produce progeny virions as in the OP7/STV co-infections experiments performed previously^4^^,^^35^ and in this work. This is similar to conventional DIPs, which cannot produce certain viral proteins due to deletions in their genome sequence and which also require complementation by STV co-infection for their propagation.^63^ Corresponding experiments aiming to confirm the defect of M1-OP7 are the subject of future studies.

The average vRNA levels in progeny virions calculated for OP7/STV co-infection showed a strong increase of S7-OP7 vRNAs, i.e., around ten copies per particle (Figures 1R and 6B), as already observed in our previous studies.^4^^,^^35^ Thus, we suspect that, besides the existence of STV particles and “7 + 1” DIPs, some progeny virions might exclusively contain copies of S7-OP7 vRNA (Figure 6). In general, S7 vRNPs are considered to play a key role during the assembly of IAV particles, because mutations to the S7 genome sequence were shown to disrupt the organized packaging of all eight genome segments.^67^^,^^68^ The SNS on S7-OP7 may also impact the organized assembly of virus particles. A potential result of such a disruption is that the available vRNAs are packaged into virions randomly^67^ according to their intracellular ratio (Figure 1F), which coincides with their extracellular ratio after co-infection (Figure 1R). This could lead to a diverse spectrum of genome compositions in progeny virions. Nevertheless, due to the sheer numbers involved (10 vRNAs/virion), it is highly likely that multiple copies of S7-OP7 vRNP accumulate in virus particles leading to virions being polyploid regarding S7-OP7 vRNA, as suggested previously.^35^

Model simulations were in good agreement with the average vRNA levels in virions and virus titers quantified during serial OP7/STV co-infection experiments demonstrating the predictive power of our OP7 model (Figure 7). These infections were mostly performed at a high multiplicity of infection (MOI), i.e., MOI 10. In this instance, nearly all cells are infected at the same time and it can be assumed that this involves at least one STV and one OP7 particle. This scenario can be described well by the single-cell model of OP7/STV co-infection we developed in this study. However, model predictions would likely be less reliable for low MOI conditions. In a low MOI infection, a mixture of STV infections, OP7 infections and OP7/STV co-infections would occur leading to a heterogeneous infection profile that cannot be captured by a single-cell model.^69^ In such a setting, a multiscale model would be required to describe the different infection scenarios.^53^

Evaluating the credible intervals of the estimated model parameters, we observed that some model parameters are correlated (Figure S5). These correlations are based on the underlying biological mechanisms of virus replication and are part of the basic structure of the model, e.g., the rate of M1 binding to vRNPs $\left(k_{M1}^{\text{Bind}}\right)$, which disables viral mRNA synthesis, is correlated to the mRNA synthesis rate $\left(k_{M}^{\text{Syn}}\right)$. However, due to the considerable amount of quantitative experimental data used for estimation, these correlations do not induce large credible intervals in the respective parameter distributions.

Overall, the model of OP7/STV co-infection closely captured virus infection dynamics induced by the mutated genomic S7-OP7 and allowed us to infer the impact of the SNS introduced to its sequence. This model could be used to describe low MOI infections by expanding it to a multiscale model that considers infection dynamics on the cell population level.^53^^,^^70^^,^^71^^,^^72^ Furthermore, it could be extended toward simulation of *in vivo* infections in tissues and organs. To that end, an expansion to the second or third spatial dimension is required.^73^^,^^74^ In addition, such a model should consider the innate immune response, which is regarded as essential for the antiviral effect of DIPs,^37^^,^^38^^,^^39^^,^^63^ especially against unrelated virus species.^40^^,^^41^^,^^42^^,^^43^ Ultimately, the co-infection model developed here is well calibrated for the prediction of IAV infections subject to OP7 interference. Thus, it provides a solid basis for the evaluation of application strategies to prevent or treat virus infections using OP7 as a novel antiviral treatment modality.

### Limitations of the study

The packaging and release of virus particles is described as a single step in our model. For this process, various interactions of cellular and viral proteins with the viral genomes are required. However, the exact mechanisms involved in this intricate process are still largely elusive. Depending on improvements to the way we understand and quantify virus release during IAV infection, these mechanisms could be described more closely in the model to evaluate the impact of the SNS on S7-OP7 packaging and release. This could also improve the description of progeny virion composition after OP7/STV co-infection. By implementing the assumption that, in addition to STV particles and regular “7 + 1” DIPs, a third kind of virus particle that contains eight copies of S7-OP7 vRNA is released, we were able to reproduce our experimental data. However, our hypothesis is only a simplifying assumption, which could be reevaluated based on further knowledge regarding virus particle release during OP7/STV co-infection.

Due to the correlations observed between some kinetic parameters, it is difficult to identify one unique parameter set from the available data. Multiple parameter combinations can describe the experimental data, which leads to the uncertainties in model prediction shown in Figure 1. However, most parameters are well constrained and are distributed similarly to previously estimated results.^53^^,^^71^ Thus, the mechanistic relationships identified in this study are strongly supported by our model-based analysis and should be evaluated experimentally in follow-up studies.

## STAR★Methods

### Key resources table

**Table:** 

REAGENT or RESOURCE	SOURCE	IDENTIFIER
**Bacterial and virus strains**		

H1N1 (A/Puerto Rico/8/34)	RKI	3138
OP7 virus	(Kupke et al.^35^)	https://doi.org/10.1128/JVI.01786-18

**Chemicals, peptides, and recombinant proteins**		

Cycloheximide (CHX)	Merck	C7698-5G
Phusion high-fidelity DNA polymerase	Thermo Scientific	F530
Maxima H Minus reverse transcriptase	Thermo Scientific	EP0752
Rotor-Gene SYBR green PCR mix	Qiagen	208056
NucleoSpin RNA kit	Macherey-Nagel	740955.250
RIPA buffer	Thermo Scientific	89900

**Critical commercial assays**		

Pierce® BCA protein assay	Thermo Scientific	23227

**Experimental models: Cell lines**		

MDCK cells	ECACC	84121903

**Software and algorithms**		

MatLab 2019b	MatLab	https://www.mathworks.com/products/matlab.html
CVODE	(Cohen et al.^75^)	https://computing.llnl.gov/projects/sundials/sundials-software
GlobalSearch	MatLab	https://www.mathworks.com/products/global-optimization.html
IQM Toolbox	(Schmidt et al.^76^)	https://iqmtools.intiquan.com/main.html
gwmcmc	(Goodman et al.^77^)	https://github.com/grinsted/gwmcmc
Skyline 19.1	(MacLean et al.^78^)	https://skyline.ms/project/home/software/skyline/begin.view
MatLab codes	This paper	https://github.com/ModIAV/OP7_STV_Coinfection

**Other**		

timsTOF Pro	Bruker	N/A
UltiMate® 3000 nano splitless reversed phase nanoHPLC	Thermo Scientific	N/A

### Resource availability

#### Lead contact

Further information and requests for resources should be directed to and will be fulfilled by the lead contact, Sascha Y. Kupke (kupke@mpi-magdeburg.mpg.de).

#### Materials availability

This study did not generate new unique reagents.

#### Data and code availability

• The experimental data are contained within the manuscript and its Supporting Information files (Data S1).
• Code used for simulation and parameter estimation is available at https://github.com/ModIAV/OP7_STV_Coinfection.
• Any additional information required to reanalyze the data reported in this paper is available from the lead contact upon request.

### Method details

#### Intracellular model of OP7/STV co-infection

The intracellular replication during OP7/STV co-infection is based on a model that was developed in our group previously.^14^ In short, this model uses a set of ordinary differential equations to describe virus binding and viral genome entry, viral RNA transcription and replication, viral protein synthesis, assembly and release of progeny virus particles for STVs and DIPs. This model was altered to describe OP7 instead of a regular DIPs with internal deletions by modifying (Equation 20), (Equation 21), (Equation 22), (Equation 23), (Equation 24), (Equation 26), (Equation 27), (Equation 28), (Equation 29), (Equation 30), (Equation 31), (Equation 32), (Equation 33), (Equation 34), 38, (Equation 40), (Equation 41), (Equation 42), and (Equation 44), (Equation 45), (Equation 46), (Equation 47), (Equation 48), (Equation 49), (Equation 50), (Equation 51), (Equation 52), (Equation 53). Therefore, we introduced S7-OP7 as the ninth genome segment considering that it has the same length as S7-STV, i.e., *L*_9_ *= L*_7_ and *L*_V,9_ = *L*_V,7_. S7-OP7 follows the same replication process as the STV segments. A detailed description of the equations and parameters representing OP7/STV co-infection is provided at the end of the STAR Methods section and in Table S3, respectively.

We introduced a reduction of mRNA transcription for S1-3, which was observed experimentally in previous studies^79^^,^^80^^,^^81^ and used to model STV and DIP co-infection successfully.^53^ To that end, we changed mRNA dynamics to

(Equation 1) $$\frac{dR_{i}^{M}}{dt}=f_{M}\frac{k_{M}^{\text{Syn}}Vp_{i}^{\text{Nuc}}}{L_{i}}-k_{M}^{\mathrm{Deg}}R_{i}^{M}$$

with,

(Equation 2) $$f_{M}=\left\{ \begin{array}{cc} F_{M}^{\text{RdRp}}, & i\in \left\{1,2,3\right\},\\ 1, & i\in \left\{4,...,9\right\}, \end{array}\right.$$

where STV mRNA of segments i = 1,…,8 and OP7 mRNA (i = 9) are synthesized with the rate $k_{M}^{\text{Syn}}$ depending on the available nuclear vRNPs $Vp_{i}^{\text{Nuc}}$ and the length of the corresponding mRNA $L_{i}$. The degradation of mRNA occurs with the rate $k_{M}^{\mathrm{Deg}}$. The transcription of S1-3 is reduced by the factor $F_{M}^{\text{RdRp}}$.

In contrast to the original intracellular DIP model, we included the synthesis of a viral protein encoded by the mutated segment. Thus, we consider two different versions of M1, i.e., M1-STV and M1-OP7,

(Equation 3) $$\frac{dP_{M1-\text{STV}}}{dt}=\frac{k_{P}^{\text{Syn}}}{D_{\text{Rib}}}\left(1-F_{\text{Spl}7}\right)R_{7}^{M}-\frac{k_{M1}^{\text{Bind}}P_{M1-\text{STV}}}{N_{M1}^{\text{Nuc}}}\sum_{i}L_{V,i}Vp_{i}^{\text{Nuc}}-\frac{P_{M1-\text{STV}}}{P_{M1,\text{Tot}}}\left[\left(N_{P_{M1}}-\frac{1}{N_{M1}^{\text{Nuc}}}\sum_{j=1,...,8}L_{V,j}\right)r_{\text{STV}}^{\text{Rel}}-\left(N_{P_{M1}}-\frac{1}{N_{M1}^{\text{Nuc}}}\sum_{g=1,...,6,8,9}L_{V,k}\right)r_{\text{DIP}}^{\text{Rel}}-\left(N_{P_{M1}}-\frac{L_{V,9}}{N_{M1}^{\text{Nuc}}}\right)r_{\text{OP}7}^{\text{Rel}}\right]$$

(Equation 4) $$\frac{dP_{M1-\text{OP}7}}{dt}=\frac{k_{P}^{\text{Syn}}}{D_{\text{Rib}}}\left(1-F_{\text{Spl}7}\right)R_{9}^{M}-\frac{k_{M1}^{\text{Bind}}P_{M1-\text{OP}7}}{N_{M1}^{\text{Nuc}}}\sum_{i}L_{V,i}Vp_{i}^{\text{Nuc}}-\frac{P_{M1-\text{OP}7}}{P_{M1,\text{Tot}}}[\left(N_{P_{M1}}-\frac{1}{N_{M1}^{\text{Nuc}}}\sum_{j=1,...,8}L_{V,j}\right)r_{\text{STV}}^{\text{Rel}}-\left(N_{P_{M1}}-\frac{1}{N_{M1}^{\text{Nuc}}}\sum_{g=1,...,6,8,9}L_{V,k}\right)r_{\text{DIP}}^{\text{Rel}}-\left(N_{P_{M1}}-\frac{L_{V,9}}{N_{M1}^{\text{Nuc}}}\right)r_{\text{OP}7}^{\text{Rel}}]$$

which are synthesized with the rate $k_{P}^{\text{Syn}}$ from their corresponding mRNAs $R_{7}^{M}$ and $R_{9}^{M}$, respectively. In the initial model implementation, both M1s can bind to vRNPs in the nucleus with the rate $k_{M1}^{\text{Bind}}$. Additionally, they are required for progeny virion release.

Moreover, we differentiated which type of M1 is binding to vRNPs in the nucleus, resulting in

(Equation 5) $$\frac{dVp_{M1-\text{STV},i}^{\text{Nuc}}}{dt}=k_{M1}^{\text{Bind}}P_{M1-\text{STV}}Vp_{i}^{\text{Nuc}}-\left(k^{\mathrm{Exp}}P_{\text{NEP}}+k_{\text{Rnp}}^{\mathrm{Deg}}\right)Vp_{M1-\text{STV},i}^{\text{Nuc}}$$

(Equation 6) $$\frac{dVp_{M1-\text{OP}7,i}^{\text{Nuc}}}{dt}=k_{M1}^{\text{Bind}}P_{M1-\text{OP}7}Vp_{i}^{\text{Nuc}}-\left(k^{\mathrm{Exp}}P_{\text{NEP}}+k_{\text{Rnp}}^{\mathrm{Deg}}\right)Vp_{M1-\text{OP}7,i}^{\text{Nuc}}$$

where $k^{\mathrm{Exp}}$ and $k_{\text{Rnp}}^{\mathrm{Deg}}$ denote the rates of export and degradation of vRNP-M1 complexes, respectively.

Furthermore, we adjusted the viral RNA replication concerning the function of RdRp. In the original DIP model, nascent vRNA and cRNA were synthesized from complementary ribonucleoprotein complexes (cRNPs) and vRNPs, respectively.^14^ These nascent RNAs were then stabilized by the binding of RdRp and, in a next step, NP to form progeny vRNPs and cRNPs. For our model of OP7/STV co-infection, we excluded the intermediate binding step mediated by RdRp. While cRNA synthesis can be catalyzed by the resident RdRp, a second RdRp likely binds to the newly synthesized cRNA right after the first nucleotides are generated.^82^ On the other hand, vRNA synthesis requires a non-resident RdRp in addition to the resident RdRp.^82^^,^^83^ Thus, we removed nascent vRNA and cRNA from the model by simplifying the dynamics of viral RNA replication to

(Equation 7) $$\frac{dR_{\text{RdRp},i}^{V}}{dt}=k_{V}^{\text{Syn}}P_{\text{RdRp}}Cp_{i}-\left(k_{\text{NP}}^{\text{Bind}}P_{\text{NP}}+k_{\text{RRdRp}}^{\mathrm{Deg}}\right)R_{\text{RdRp},i}^{V}$$

(Equation 8) $$\frac{dR_{\text{RdRp},i}^{C}}{dt}=k_{C}^{\text{Syn}}P_{\text{RdRp}}Vp_{i}^{\text{Nuc}}-\left(k_{\text{NP}}^{\text{Bind}}P_{\text{NP}}+k_{\text{RRdRp}}^{\mathrm{Deg}}\right)R_{\text{RdRp},i}^{C}$$

where $R_{\text{RdRp},i}^{V}$ and $R_{\text{RdRp},i}^{C}$ represent vRNA and cRNA bound by a single RdRp. They are synthesized from cRNPs ($Cp_{i}$) and vRNPs with the rates $k_{V}^{\text{Syn}}$ and $k_{C}^{\text{Syn}}$, respectively. Then, they can either be stabilized by NP with the rate $k_{\text{NP}}^{\text{Bind}}$ or degrade with the rate $k_{\text{RRdRp}}^{\mathrm{Deg}}$.

#### Model extension

We extended this modified model to describe the virus infection dynamics observed during OP7/STV co-infection. As in the original model, we included a replication advantage for the defective segment (Equations 24 and 27). In addition, we considered a decreased transcription of S7-OP7 mRNA induced by the SNS

(Equation 9) $$\frac{dR_{9}^{M}}{dt}=\left(1-F_{M}^{\text{OP}7}\right)\frac{k_{M}^{\text{Syn}}Vp_{9}^{\text{Nuc}}}{L_{9}}-k_{M}^{\mathrm{Deg}}R_{9}^{M}$$

where $F_{M}^{\text{OP}7}$ represents the factor by which the synthesis of S7-OP7 mRNA is reduced.

Furthermore, we considered M1-OP7 to be defective regarding the binding of vRNPs in the nucleus. Thus, vRNPs can only be bound by M1-STV and the differential equation for M1-OP7 changes to

(Equation 10) $$\frac{dP_{M1-\text{OP}7}}{dt}=\frac{k_{P}^{\text{Syn}}}{D_{\text{Rib}}}\left(1-F_{\text{Spl}7}\right)R_{9}^{M}-\frac{P_{M1-\text{OP}7}}{P_{M1,\text{Tot}}}[\left(N_{P_{M1}}-\frac{1}{N_{M1}^{\text{Nuc}}}\sum_{j=1,...,8}L_{V,j}\right)r_{\text{STV}}^{\text{Rel}}-\left(N_{P_{M1}}-\frac{1}{N_{M1}^{\text{Nuc}}}\sum_{g=1,...,6,8,9}L_{V,k}\right)r_{\text{DIP}}^{\text{Rel}}-\left(N_{P_{M1}}-\frac{L_{V,9}}{N_{M1}^{\text{Nuc}}}\right)r_{\text{OP}7}^{\text{Rel}}]$$

Additionally, the equation describing vRNPs bound by M1-OP7 (Equation 6) is not required anymore.

Lastly, we introduced a third type of progeny virus particle that only contains S7-OP7. For this particle, we did not consider the formation of S7-OP7 complexes prior to release. We also assumed that multiple copies of S7-OP7 vRNP can be packaged into virions. Thus, the release of these new particles is described by

(Equation 11) $$\frac{d\text{OP}7^{\text{Rel}}}{dt}=r_{\text{OP}7}^{\text{Rel}}=k^{\text{Rel}}\frac{Vp_{M1,9}^{\text{Cyt}}}{V_{\text{Cplx}}^{\text{Cyt}}+D_{\text{Cplx}}^{\text{Cyt}}+Vp_{M1,9}^{\text{Cyt}}+K_{V^{\text{Rel}}}}\prod_{p}\frac{P_{p}}{P_{p}+N_{P_{p}}K_{V^{\text{Rel}}}}$$

where S7-OP7 vRNPs in the cytoplasm $Vp_{M1,9}^{\text{Cyt}}$ are released utilizing the viral proteins p ∈ {HA, NA, M1, M2} with the rate $k^{\text{Rel}}$. This rate represents a maximum release of virus particles and is distributed among STV complexes ($V_{\text{Cplx}}^{\text{Cyt}}$), “7+1” DIP-like complexes ($D_{\text{Cplx}}^{\text{Cyt}}$) and S7-OP7 vRNPs. To obtain the actual number of OP7 particles, $r_{\text{OP}7}^{\text{Rel}}$ can be divided by the average amount of S7-OP7 vRNPs per OP7 particles, i.e., $N_{\text{OP}7}^{\text{Par}}$. For the sake of simplicity, we assumed this is analogous to the number of vRNPs in an STV particle resulting in $N_{\text{OP}7}^{\text{Par}}=8\;\text{molecules}$.

#### Simulation approach and parameter estimation

In general, model simulations were performed according to a previously published model of STV and DIP co-infection.^14^ Model equations were solved numerically with the CVODE routine from SUNDIALS^75^ on a Linux-based system. Experimental data and model files were processed with the Systems Biology Toolbox 2 for MatLab (version 9.7.0.1296695, R2019b).^76^

Baseline model parameters were calibrated by fitting the OP7/STV co-infection model to infection dynamics measured during an STV infection (Figures 1A–1C, 1G–1I, 1M, and 1N). The replication advantage $F_{C,V}^{\mathrm{Adv}}$ and the reduced transcription $F_{M}^{\text{OP7}}$ of S7-OP7 were determined by fitting the model to experimental data obtained from an OP7/STV co-infection (Figures 1D–1F, 1J–1L, 1P, and 1Q). The global optimization algorithm *GlobalSearch*, which is implemented in MatLab, was employed to determine optimal sets of parameters. For the calculation of the sum of squared residuals, experimental data measured at *t* = 0 were added as an offset for viral RNAs and proteins to accommodate for a background signal in the RT-qPCR and mass spectrometry analysis, respectively. All parameter values used for model simulation are shown in Table S3.

Credible intervals in Table S1 were determined via Bayesian inference using the affine invariant ensemble Markov Chain Monte Carlo (MCMC) sampler developed by Goodman and Weare.^77^^,^^84^ Employing the MCMC implementation for MatLab (*gwmcmc*), we obtained the parameter distributions presented in Figures S5 and S6. Uniformly distributed priors were assumed for the estimated parameters based on the final parameter sets determined by the global optimization algorithm. Posterior distributions were constructed from 2,000,000 and 500,000 samples when fitting to STV infection and OP7/STV co-infection data, respectively. We applied a burn-in phase, which is performed to reach an assumed equilibrium distribution before sampling, by discarding the first 25% of results obtained. Additionally, we performed a thinning step by retaining every eighth sample to reduce the correlation between individual samples. Thus, the resulting parameter distributions contain 187,500 and 46,875 samples for the fits to the STV infection and OP7/STV co-infection data, respectively.

The prediction bands shown in Figure 1 are based on the 95% credible intervals of 50,000 simulations using parameter values sampled from the parameter distributions shown in Figures S5 and S6.

#### Model prediction

For the prediction of infection passages starting at different initial concentrations of S7-OP7 vRNA, we simulated the OP7/STV co-infection model using the parameters calibrated to our experimental data. Simulation of the first passages was initiated with the experimentally determined vRNA concentrations (Figures 7A–7D). For further passages, the vRNA levels in progeny virions at 12 hpi, which were predicted for the previous passage, were applied as the initial condition (Figures 7E–7L). According to the experimental set-up, an MOI of 10 was used to simulate the predictions shown in Figures 7E–7K. For the prediction in Figure 7L, an MOI of 1 was employed.

#### Cells and viruses

Adherent MDCK cells (ECACC, #84121903) were cultivated in Glasgow minimum essential medium (GMEM) containing 10% fetal bovine serum (FBS) and 1% peptone at 5% CO_2_ and 37°C. Influenza A virus strain PR/8/34 (PR8) of subtype H1N1 was used for STV infections and was obtained from the Robert Koch Institute Germany (#3138). OP7 virions were enriched to yield a seed virus containing a mixture of STV and OP7 particles from PR8 provided by the National Institute for Biological Standards and Control (#99/716) as described previously.^35^ For virus infection, a serum-free medium was used.

Seed virus titers and infectious virus titers were determined by a standard TCID_50_ assay using adherent MDCK cells.^85^ MOIs applied for infection experiments were based on this titer. The total virus particle titer was measured via HA assay,^86^ expressed as log_10_ HA units per test volume (log HAU/100 μL). The concentration of total virus particles was calculated as described in Burleson et al.^87^ according to

(Equation 12) $$c_{\text{Virus}}=2\times 10^{7}\times 10^{\left(\log\text{HAU}/100\;\mu L\right)}$$

Serial OP7/STV co-infection passages were performed based on a standard infection protocol described in Kupke et al.^35^

#### Determination of viral mRNA accumulation rates

Adherent MDCK cells cultivated in 6-well plates were washed with phosphate-buffered saline (PBS) and incubated in medium (GMEM, 1% peptone) containing CHX (#C7698-5G, Merck) at a final concentration of 100 μg/ml for 1 h prior to infection. Next, cells were either infected with PR8 STV or OP7 seed virus (MOI 10) in 250 μl of infection medium (GMEM, 1% peptone, 5 U/ml trypsin) containing CHX (100 μg/ml), and incubated for 1 h at 37°C, 5% CO2. Subsequently, the inoculum was removed, the cells were washed with PBS, and 2 ml of infection medium containing CHX was added. Cells maintained and infected in the corresponding CHX-free media were used as controls. For each investigated time point, one individual well was used. The supernatant was removed and the cells were harvested for RNA isolation. Cell lysis and intracellular RNA extraction were performed using the NucleoSpin RNA kit (#740955.250, Macherey-Nagel) according to the manufacturer’s instructions. Real-time RT-qPCR was used to quantify mRNAs and vRNAs of S5, S7-STV, S7-OP7, and S8 in the isolated material. Calculations of viral RNA levels per cell were based on the cell count at the time of infection.

#### Real-time RT-qPCR to quantify viral RNAs

Samples originating from the infection experiments performed in Kupke et al.^35^ were analyzed via real-time RT-qPCR to quantify (i) the segment-specific concentrations of vRNA of progeny virions at 12 hpi and (ii) the dynamics of the segment-specific levels of mRNA, cRNA and vRNA per cell. The analysis was performed according to the protocol described in Kupke et al.^35^ To differentiate between the RNAs of S7-STV and S7-OP7, the set of primers was adjusted compared to the original publication (Tables S4–S6). The primers for S5 and S8 RNAs were taken from Kupke et al.,^35^ for S7-OP7 vRNA from Hein et al.,^4^ and for S7-STV vRNA from Dogra et al.^88^ The primers for S7-OP7 and S7-STV mRNA and cRNA were designed specifically for this study.

The vRNA levels measured in progeny virions were normalized twice to obtain a baseline of one copy of each STV vRNA segment per virus particle during a regular STV infection (Figure 1O). First, the values were normalized to the total virus particle concentration obtained via HA assay. Then, they were normalized to the average vRNA levels in virions during an STV infection. For S7-OP7, the reference value of S7-STV from the STV infection was applied.

#### Quantification of influenza virus proteins

Influenza proteins were analyzed via mass spectrometry.^56^ Absolute protein copy numbers were determined for M1-STV, M1-OP7, NP, and PB1 for samples originating from STV infection and OP7/STV co-infection. For analysis of intracellular proteins, cell pellet samples were lysed via RIPA buffer (#89900, Thermo Scientific). Then, total protein concentration was determined by using Pierce® BCA protein assay (#23227, Thermo Scientific) according to the manufacturer’s protocol. For quantification of total extracellular protein, BCA assay was used on supernatant samples. Then, preparation for mass spectrometry measurements was carried out with 50 μg protein of intra- or extracellular samples by using the UlitMate3000 nano-LC system coupled with a timsTOF pro mass spectrometer.^56^

Determination of absolute protein copy numbers was facilitated by adding synthetically labelled peptides of the analyzed proteins to the samples. For quantification of M1-STV, two peptides, which are mutated in M1-OP7 and only occur in M1-STV, were measured. To differentiate M1-STV from M1-OP7, a peptide containing one nucleotide substitution of S7-OP7 was measured to quantify M1-OP7 specifically.^56^^,^^88^

Further processing, measurement and analysis were carried out with the open source software Skyline^78^ as described in Dogra et al.^88^

#### Full model of OP7/STV co-infection

##### Virus entry

(Equation 13) $$\frac{dV^{\text{Ex}}}{dt}=k_{Hi}^{\text{Dis}}V_{\text{Hi}}^{\text{Att}}+k_{\text{Lo}}^{\text{Dis}}V_{\text{Lo}}^{\text{Att}}-\left(k_{\text{Hi}}^{\text{Att}}B_{\text{Hi}}+k_{\text{Lo}}^{\text{Att}}B_{\text{Lo}}\right)V^{\text{Ex}}$$

with,

(Equation 14) $$B_{n}=B_{n}^{\text{Tot}}-V_{n}^{\text{Att}},n\in \left\{\text{Hi},\text{Lo}\right\}$$

and,

(Equation 15) $$k_{n}^{\text{Dis}}=\frac{k_{n}^{\text{Att}}}{k_{n}^{\text{Eq}}}$$

(Equation 16) $$\frac{dV_{n}^{\text{Att}}}{dt}=k_{n}^{\text{Att}}B_{n}V^{\text{Ex}}-\left(k_{n}^{\text{Dis}}+k^{\text{En}}\right)V_{n}^{\text{Att}}$$

(Equation 17) $$\frac{dV^{\text{En}}}{dt}=k^{\text{En}}\left(V_{\text{Hi}}^{\text{Att}}+V_{\text{Lo}}^{\text{Att}}\right)-\left(k^{\text{Fus}}+k_{\text{En}}^{\mathrm{Deg}}\right)V^{\text{En}}$$

with

(Equation 18) $$k_{\text{En}}^{\mathrm{Deg}}=\frac{1-F_{\text{Fus}}}{F_{\text{Fus}}}k^{\text{Fus}},0<F_{\text{Fus}}\leq 1$$

$V^{\text{Ex}}$ represents extracellular virus particles that bind to free high- and low-affinity binding sites ($B_{n}$) on the cell membrane.^89^^,^^90^ For virus entry, different types of infecting virions, i.e., STV particles, “7+1” DIPs and OP7 particles, are considered. However, they are not described as separate states. Instead, they are conflated in $V^{\text{Ex}}$ and the average amount of individual genome segments, i.e., STV segments 1 to 8 (S1-8) and S7-OP7, per infecting particle is considered. With this, we assume that the different particles follow the same entry mechanism. Virions attached to binding sites ($V_{n}^{\text{Att}}$) either dissociate from the cell membrane or undergo receptor-mediated endocytosis with rates $k_{n}^{\text{Dis}}$ and $k^{\text{En}}$, respectively. Then, enveloped virus particles ($V^{\text{En}}$) can transfer their viral genome into the cytoplasm or degrade in lysosomes.

##### Virus replication

(Equation 19) $$\frac{dV^{\text{Cyt}}}{dt}=k^{\text{Fus}}V^{\text{En}}-k^{\text{Imp}}V^{\text{Cyt}}$$

(Equation 20) $$\frac{dVp_{k}^{\text{Nuc}}}{dt}=k^{\text{Imp}}N_{\text{STV}}^{\text{In}}V^{\text{Cyt}}+k_{\text{NP}}^{\text{Bind}}P_{\text{NP}}R_{\text{RdRp},k}^{V}-\left(k_{M1}^{\text{Bind}}P_{M1-\text{STV}}+k_{\text{Rnp}}^{\mathrm{Deg}}\right)Vp_{k}^{\text{Nuc}}$$

(Equation 21) $$\frac{dVp_{7}^{\text{Nuc}}}{dt}=k^{\text{Imp}}N_{S7-\text{STV}}^{\text{In}}V^{\text{Cyt}}+k_{\text{NP}}^{\text{Bind}}P_{\text{NP}}R_{\text{RdRp},7}^{V}-\left(k_{M1}^{\text{Bind}}P_{M1-\text{STV}}+k_{\text{Rnp}}^{\mathrm{Deg}}\right)Vp_{7}^{\text{Nuc}}$$

(Equation 22) $$\frac{dVp_{9}^{\text{Nuc}}}{dt}=k^{\text{Imp}}N_{S7-\text{OP}7}^{\text{In}}V^{\text{Cyt}}+k_{\text{NP}}^{\text{Bind}}P_{\text{NP}}R_{\text{RdRp},9}^{V}-\left(k_{M1}^{\text{Bind}}P_{M1-\text{STV}}+k_{\text{Rnp}}^{\mathrm{Deg}}\right)Vp_{9}^{\text{Nuc}}$$

(Equation 23) $$\frac{dR_{\text{RdRp},j}^{C}}{dt}=k_{C}^{\text{Syn}}P_{\text{RdRp}}Vp_{j}^{\text{Nuc}}-\left(k_{\text{NP}}^{\text{Bind}}P_{\text{NP}}+k_{\text{RRdRp}}^{\mathrm{Deg}}\right)R_{\text{RdRp},j}^{C}$$

(Equation 24) $$\frac{dR_{\text{RdRp},9}^{C}}{dt}=\left(1+F_{C,V}^{\text{Adv}}\right)k_{C}^{\text{Syn}}P_{\text{RdRp}}Vp_{9}^{\text{Nuc}}-\left(k_{\text{NP}}^{\text{Bind}}P_{\text{NP}}+k_{\text{RRdRp}}^{\mathrm{Deg}}\right)R_{\text{RdRp},9}^{C}$$

(Equation 25) $$\frac{dCp_{i}}{dt}=k_{\text{NP}}^{\text{Bind}}P_{\text{NP}}R_{\text{RdRp},i}^{C}-k_{\text{Rnp}}^{\mathrm{Deg}}Cp_{i}$$

(Equation 26) $$\frac{dR_{\text{RdRp},j}^{V}}{dt}=k_{V}^{\text{Syn}}P_{\text{RdRp}}Cp_{j}-\left(k_{\text{NP}}^{\text{Bind}}P_{\text{NP}}+k_{\text{RRdRp}}^{\mathrm{Deg}}\right)R_{\text{RdRp},j}^{V}$$

(Equation 27) $$\frac{dR_{\text{RdRp},9}^{V}}{dt}=\left(1+F_{C,V}^{\text{Adv}}\right)k_{V}^{\text{Syn}}P_{\text{RdRp}}Cp_{9}-\left(k_{\text{NP}}^{\text{Bind}}P_{\text{NP}}+k_{\text{RRdRp}}^{\mathrm{Deg}}\right)R_{\text{RdRp},9}^{V}$$

(Equation 28) $$\frac{dVp_{M1,i}^{\text{Nuc}}}{dt}=k_{M1}^{\text{Bind}}P_{M1-\text{STV}}Vp_{i}^{\text{Nuc}}-\left(k^{\mathrm{Exp}}P_{\text{NEP}}+k_{\text{Rnp}}^{\mathrm{Deg}}\right)Vp_{M1,i}^{\text{Nuc}}$$

(Equation 29) $$\frac{dVp_{M1,k}^{\text{Cyt}}}{dt}=k^{\mathrm{Exp}}P_{\text{NEP}}Vp_{M1,k}^{\text{Nuc}}-k_{\text{Rnp}}^{\mathrm{Deg}}Vp_{M1,k}^{\text{Cyt}}-k^{\text{Cplx}}\left(Vp_{M1,7}^{\text{Cyt}}+Vp_{M1,9}^{\text{Cyt}}\right)\prod_{g=1,...,6,8}Vp_{M1,g}^{\text{Cyt}}$$

(Equation 30) $$\frac{dVp_{M1,7}^{\text{Cyt}}}{dt}=k^{\mathrm{Exp}}P_{\text{NEP}}Vp_{M1,7}^{\text{Nuc}}-k_{\text{Rnp}}^{\mathrm{Deg}}Vp_{M1,7}^{\text{Cyt}}-k^{\text{Cplx}}Vp_{M1,7}^{\text{Cyt}}\prod_{g=1,...,6,8}Vp_{M1,g}^{\text{Cyt}}$$

(Equation 31) $$\frac{dVp_{M1,9}^{\text{Cyt}}}{dt}=k^{\mathrm{Exp}}P_{\text{NEP}}Vp_{M1,9}^{\text{Nuc}}-k_{\text{Rnp}}^{\mathrm{Deg}}Vp_{M1,9}^{\text{Cyt}}-k^{\text{Cplx}}Vp_{M1,9}^{\text{Cyt}}\prod_{g=1,...,6,8}Vp_{M1,g}^{\text{Cyt}}-k^{\text{Rel}}\frac{Vp_{M1,9}^{\text{Cyt}}}{V_{\text{Cplx}}^{\text{Cyt}}+D_{\text{Cplx}}^{\text{Cyt}}+Vp_{M1,9}^{\text{Cyt}}+K_{V^{\text{Rel}}}}\prod_{p}\frac{P_{p}}{P_{p}+N_{P_{p}}K_{V^{\text{Rel}}}}$$

with i = 1,…,9; j = 1,…,8; k = 1,…,6,8 and p ∈ {HA, NA, M1, M2}.

Subsequently, viral ribonucleoproteins (vRNPs) in the cytoplasm ($V^{\text{Cyt}}$) are imported into the nucleus. Here, the composition of the seed virus regarding different types of virus particles is considered via the average amount of viral genomic RNA (vRNA) per virion of S7-STV, S7-OP7 and the other STV segments ($N_{S7-\text{STV}}^{\text{In}}$, $N_{S7-\text{OP}7}^{\text{In}}$, $N_{\text{STV}}^{\text{In}}$). In the nucleus, the vRNPs of different viral genome segments ($Vp_{i}^{\text{Nuc}}$) are used as templates for virus replication, with i = 1,…,8 denoting the eight STV segments and i = 9 representing S7-OP7. Complementary RNA (cRNA) $R_{\text{RdRp},i}^{C}$ is transcribed from nuclear vRNPs by viral RNA-dependent-RNA-polymerase (RdRp) $P_{\text{RdRp}}$ and subsequently stabilized by binding nucleoproteins (NP) $P_{\text{NP}}$ to form complementary ribonucleoproteins (cRNP) $Cp_{i}$. Then, progeny vRNA is transcribed from the cRNP templates by RdRp and bound by NP forming $R_{\text{RdRp}.i}^{V}$ and $Vp_{i}^{\text{Nuc}}$, respectively. We assume that S7-OP7 cRNA and vRNA show an enhanced replication compared to the STV segments, which is implemented in Equations 24 and 27 via the (advantage) factor $F_{C,V}^{\text{Adv}}$. The viral matrix protein 1 (M1) derived from S7-STV (M1-STV) $P_{M1-\text{STV}}$ can bind to vRNPs in the nucleus to form vRNP-M1 complexes ($Vp_{M1,i}^{\text{Nuc}}$), which are replication-incompetent. We assume the M1 derived from S7-OP7 (M1-OP7) $P_{M1-\text{OP}7}$ is defective and does not bind to vRNPs in the nucleus. Then, the nuclear export protein (NEP) $P_{\text{NEP}}$ can bind to the vRNP-M1 complexes, which enables their export to the cytoplasm where they are referred to as $Vp_{M1,i}^{\text{Cyt}}$.

##### V**iral transcription and protein synthesis**

(Equation 32) $$\frac{dR_{j}^{M}}{dt}=f_{M}\frac{k_{M}^{\text{Syn}}Vp_{j}^{\text{Nuc}}}{L_{j}}-k_{M}^{\mathrm{Deg}}R_{j}^{M}$$

with

(Equation 33) $$f_{M}=\left\{ \begin{array}{cc} F_{M}^{\text{RdRp}}, & j\in \left\{1,2,3\right\},\\ 1, & \;j\in \left\{4,...,9\right\}, \end{array}\right.$$

(Equation 34) $$\frac{dR_{9}^{M}}{dt}=\left(1-F_{M}^{\text{OP}7}\right)\frac{k_{M}^{\text{Syn}}Vp_{9}^{\text{Nuc}}}{L_{9}}-k_{M}^{\mathrm{Deg}}R_{9}^{M}$$

(Equation 35) $$\frac{dP_{\text{PB}1}}{dt}=\frac{k_{P}^{\text{Syn}}}{D_{\text{Rib}}}R_{2}^{M}-k^{\text{RdRp}}P_{\text{PB}1}P_{\text{PB}2}P_{\text{PA}}$$

(Equation 36) $$\frac{dP_{\text{PB}2}}{dt}=\frac{k_{P}^{\text{Syn}}}{D_{\text{Rib}}}R_{1}^{M}-k^{\text{RdRp}}P_{\text{PB}1}P_{\text{PB}2}P_{\text{PA}}$$

(Equation 37) $$\frac{dP_{\text{PA}}}{dt}=\frac{k_{P}^{\text{Syn}}}{D_{\text{Rib}}}R_{3}^{M}-k^{\text{RdRp}}P_{\text{PB}1}P_{\text{PB}2}P_{\text{PA}}$$

(Equation 38) $$\frac{dP_{\text{RdRp}}}{dt}=k^{\text{RdRp}}P_{\text{PB}1}P_{\text{PB}2}P_{\text{PA}}-k_{V}^{\text{Syn}}P_{\text{RdRp}}\sum_{i}\left(Cp_{i}\right)-k_{C}^{\text{Syn}}P_{\text{RdRp}}\sum_{i}\left(Vp_{i}^{\text{Nuc}}\right)$$

(Equation 39) $$\frac{dP_{\text{NP}}}{dt}=\frac{k_{P}^{\text{Syn}}}{D_{\text{Rib}}}R_{5}^{M}-\frac{k_{\text{NP}}^{\text{Bind}}P_{\text{NP}}}{N_{\text{NP}}^{\text{Nuc}}}\sum_{i}L_{V,i}\left(R_{\text{RdRp},i}^{V}+R_{\text{RdRp},i}^{C}\right)$$

(Equation 40) $$\frac{dP_{M1-\text{STV}}}{dt}=\frac{k_{P}^{\text{Syn}}}{D_{\text{Rib}}}\left(1-F_{\text{Spl}7}\right)R_{7}^{M}-\frac{k_{M1}^{\text{Bind}}P_{M1-\text{STV}}}{N_{M1}^{\text{Nuc}}}\sum_{i}\left(L_{V,i}Vp_{i}^{\text{Nuc}}\right)-\frac{P_{M1-\text{STV}}}{P_{M1,\text{Tot}}}\left[\left(N_{P_{M1}}-\frac{1}{N_{M1}^{\text{Nuc}}}\sum_{j}L_{V,j}\right)r_{\text{STV}}^{\text{Rel}}-\left(N_{P_{M1}}-\frac{1}{N_{M1}^{\text{Nuc}}}\sum_{g=1,...,6,8,9}L_{V,g}\right)r_{\text{DIP}}^{\text{Rel}}-\left(N_{P_{M1}}-\frac{L_{V,9}}{N_{M1}^{\text{Nuc}}}\right)r_{\text{OP}7}^{\text{Rel}}\right]$$

(Equation 41) $$\frac{dP_{M1-\text{OP}7}}{dt}=\frac{k_{P}^{\text{Syn}}}{D_{\text{Rib}}}\left(1-F_{\text{Spl}7}\right)R_{9}^{M}-\frac{P_{M1-\text{OP}7}}{P_{M1,\text{Tot}}}[\left(N_{P_{M1}}-\frac{1}{N_{M1}^{\text{Nuc}}}\sum_{j}L_{V,j}\right)r_{\text{STV}}^{\text{Rel}}-\left(N_{P_{M1}}-\frac{1}{N_{M1}^{\text{Nuc}}}\sum_{g=1,...,6,8,9}L_{V,g}\right)r_{\text{DIP}}^{\text{Rel}}-\left(N_{P_{M1}}-\frac{L_{V,9}}{N_{M1}^{\text{Nuc}}}\right)r_{\text{OP}7}^{\text{Rel}}]$$

with

(Equation 42) $$P_{M1,\text{Tot}}=P_{M1-\text{STV}}+P_{M1-\text{OP}7}$$

(Equation 43) $$\frac{dP_{\text{NEP}}}{dt}=\frac{k_{P}^{\text{Syn}}}{D_{\text{Rib}}}F_{\text{Spl}8}R_{8}^{M}-k^{\mathrm{Exp}}P_{\text{NEP}}\sum_{i}Vp_{M1,i}^{\text{Nuc}}$$

(Equation 44) $$\frac{dP_{\text{HA}}}{dt}=\frac{k_{P}^{\text{Syn}}}{D_{\text{Rib}}}R_{4}^{M}-N_{P_{\text{HA}}}\left(r_{\text{STV}}^{\text{Rel}}+r_{\text{DIP}}^{\text{Rel}}+\frac{r_{\text{OP}7}^{\text{Rel}}}{N_{\text{OP}7}^{\text{Par}}}\right)$$

(Equation 45) $$\frac{dP_{\text{NA}}}{dt}=\frac{k_{P}^{\text{Syn}}}{D_{\text{Rib}}}R_{6}^{M}-N_{P_{\text{NA}}}\left(r_{\text{STV}}^{\text{Rel}}+r_{\text{DIP}}^{\text{Rel}}+\frac{r_{\text{OP}7}^{\text{Rel}}}{N_{\text{OP}7}^{\text{Par}}}\right)$$

(Equation 46) $$\frac{dP_{M2}}{dt}=\frac{k_{P}^{\text{Syn}}}{D_{\text{Rib}}}F_{\text{Spl}7}\left(R_{7}^{M}+R_{9}^{M}\right)-N_{P_{M2}}\left(r_{\text{STV}}^{\text{Rel}}+r_{\text{DIP}}^{\text{Rel}}+\frac{r_{\text{OP}7}^{\text{Rel}}}{N_{\text{OP}7}^{\text{Par}}}\right)$$

Nuclear vRNPs are also used as templates for the transcription of viral messenger RNA (mRNA) $R_{i}^{M}$ and each of the genome segments encodes for a different mRNA. The transcription of mRNAs depends on their respective length ($L_{i}$) and is reduced for segments encoding for RdRp-related proteins, i.e., STV S1 to S3, by the factor $F_{M}^{\text{RdRp}}$. Additionally, the generation of S7-OP7 mRNA is reduced by the factor $F_{M}^{\text{OP}7}$ to take into account the impact of the single-nucleotide substitutions (SNSs) on OP7 transcription. mRNAs are degraded with the rate $k_{M}^{\mathrm{Deg}}$ and translated into viral proteins $P_{i}$ in the cytoplasm. The polymerase sub-unit proteins ($P_{\text{PB}1}$, $P_{\text{PB}2}$ and $P_{\text{PA}}$) bind to form RdRp. The mRNA of S7 can be translated into two different proteins, i.e., M1 and M2, and their production ratio is defined by $F_{\text{Spl}7}$. S7-STV and S7-OP7 are translated into two different M1s ($P_{M1-\text{STV}}$ and $P_{M1-\text{OP}7}$) and only M1-STV can bind vRNPs in the nucleus. However, we assume both S7-STV and S7-OP7 can be translated into regular M2. The proteins M1-STV, M1-OP7, M2, hemagglutinin (HA) and neuraminidase (NA) are required for virion release as they perform structural functions in progeny virions.

##### Complex formation and virus particle release

(Equation 47) $$\frac{dV_{\text{Cplx}}^{\text{Cyt}}}{dt}=k^{\text{Cplx}}Vp_{M1,7}^{\text{Cyt}}\prod_{g=1,...,6,8}\left(Vp_{M1,g}^{\text{Cyt}}\right)-r_{\text{STV}}^{\text{Rel}}-k_{\text{Rnp}}^{\mathrm{Deg}}V_{\text{Cplx}}^{\text{Cyt}}$$

(Equation 48) $$\frac{dD_{\text{Cplx}}^{\text{Cyt}}}{dt}=k^{\text{Cplx}}Vp_{M1,9}^{\text{Cyt}}\prod_{g=1,...,6,8}\left(Vp_{M1,g}^{\text{Cyt}}\right)-r_{\text{DIP}}^{\text{Rel}}-k_{\text{Rnp}}^{\mathrm{Deg}}D_{\text{Cplx}}^{\text{Cyt}}$$

(Equation 49) $$\frac{dV^{\text{Rel}}}{dt}=r_{\text{STV}}^{\text{Rel}}=k^{\text{Rel}}\frac{V_{\text{Cplx}}^{\text{Cyt}}}{V_{\text{Cplx}}^{\text{Cyt}}+D_{\text{Cplx}}^{\text{Cyt}}+V_{M1,9}^{\text{Cyt}}+K_{V^{\text{Rel}}}}\prod_{p}\frac{P_{p}}{P_{p}+N_{P_{p}}K_{V^{\text{Rel}}}}$$

(Equation 50) $$\frac{dD^{\text{Rel}}}{dt}=r_{\text{DIP}}^{\text{Rel}}=k^{\text{Rel}}\frac{D_{\text{Cplx}}^{\text{Cyt}}}{V_{\text{Cplx}}^{\text{Cyt}}+D_{\text{Cplx}}^{\text{Cyt}}+V_{M1,9}^{\text{Cyt}}+K_{V^{\text{Rel}}}}\prod_{p}\frac{P_{p}}{P_{p}+N_{P_{p}}K_{V^{\text{Rel}}}}$$

(Equation 51) $$\frac{d\text{OP}7^{\text{Rel}}}{dt}=r_{\text{OP}7}^{\text{Rel}}=k^{\text{Rel}}\frac{Vp_{M1,9}^{\text{Cyt}}}{V_{\text{Cplx}}^{\text{Cyt}}+D_{\text{Cplx}}^{\text{Cyt}}+Vp_{M1,9}^{\text{Cyt}}+K_{V^{\text{Rel}}}}\prod_{p}\frac{P_{p}}{P_{p}+N_{P_{p}}K_{V^{\text{Rel}}}}$$

with p∈{HA,NA,M1,M2}

(Equation 52) $$\frac{dV_{\mathrm{Inf}}^{\text{Rel}}}{dt}=F_{\text{Par}}r_{\text{STV}}^{\text{Rel}}$$

(Equation 53) $$\frac{dV_{\text{Tot}}^{\text{Rel}}}{dt}=r_{\text{STV}}^{\text{Rel}}+r_{\text{DIP}}^{\text{Rel}}+\frac{r_{\text{OP}7}^{\text{Rel}}}{N_{\text{OP}7}^{\text{Par}}}$$

The packaging of vRNPs in the cytoplasm is represented by a segment-specific mechanism. Cytoplasmic vRNPs form complexes including one copy of each genome segment. STV and “7+1” DIP complexes ($V_{\text{Cplx}}^{\text{Cyt}}$, $D_{\text{Cplx}}^{\text{Cyt}}$) both contain STV S1-6 and S8. In addition, they carry either a S7-STV or a S7-OP7 vRNP. Complex formation occurs with rate $k^{\text{Cplx}}$ and degradation with rate $k_{\text{Rnp}}^{\mathrm{Deg}}$. In total, we consider the release of three different virus particles, i.e., STV particles, “7+1” DIPs and particles that only contain S7-OP7. Their release is described by the rates $r_{\text{STV}}^{\text{Rel}}$, $r_{\text{DIP}}^{\text{Rel}}$ and $r_{\text{OP}7}^{\text{Rel}}$, respectively. These rates are calculated considering the maximum release rate $k^{\text{Rel}}$, the available vRNP complexes and the abundance of viral proteins. Furthermore, we use $V_{\mathrm{Inf}}^{\text{Rel}}$ and $V_{\text{Tot}}^{\text{Rel}}$ to represent the generated amount of infectious STV particles and the total amount of virions, respectively. The percentage of fully functional STVs is determined by the parameter $F_{\text{Par}}$, which assumes that only a fraction of produced STV particles is infectious.^71^ To calculate the absolute amount of released OP7 particles, $r_{\text{OP}7}^{\text{Rel}}$ is divided by the amount of S7-OP7 vRNPs included in one OP7 particle, i.e., $N_{\text{OP}7}^{\text{Par}}$. Here, we assume that $N_{\text{OP}7}^{\text{Par}}=8\;\text{molecules}$, analogous to the number of vRNPs in a STV particle.

## Supplemental information

Document S1. Figures S1–S6 and Tables S1–S6

Data S1. Experimental data used for model calibration, related to Figure 1
