# Supplementary material for: Mathematical model calibrated to in vitro data predicts mechanisms of antiviral action of the influenza defective interfering particle “OP7”
Source: iScience. 2024 Mar 5;27(4):109421. doi: 10.1016/j.isci.2024.109421 (PMC10959662; doi:10.1016/j.isci.2024.109421)
Supplement: Document S1. Figures S1–S6 and Tables S1–S6 [file mmc1.pdf]

iScience, Volume *27*

Supplemental information

Mathematical model calibrated to *in vitro* data predicts mechanisms of antiviral action of the influenza defective interfering particle "OP7"

Daniel Rüdiger, Julita Piasecka, Jan Küchler, Carolina Pontes, Tanja Laske, Sascha Y. Kupke, and Udo Reichl

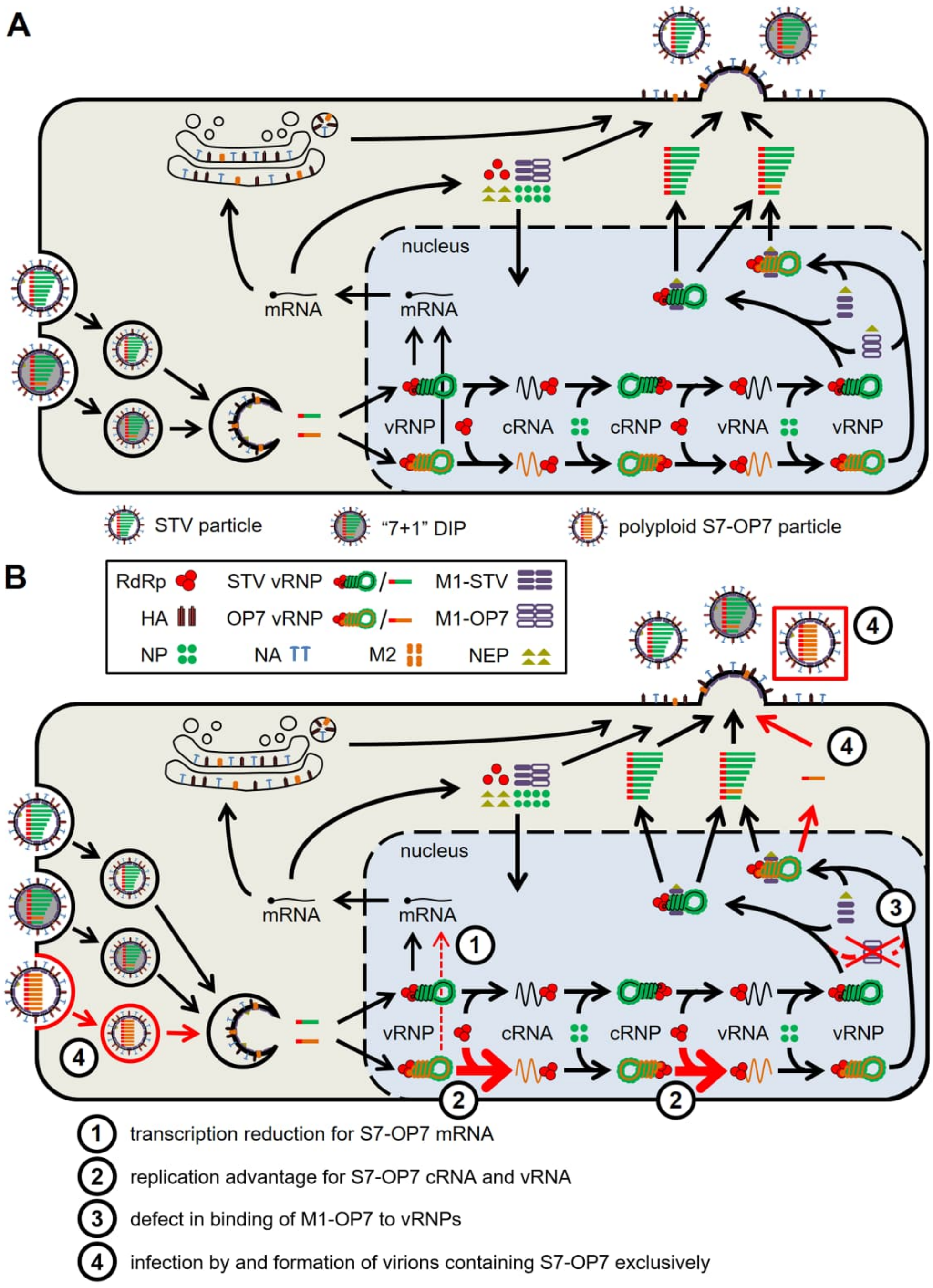

Figure S1. Visualization of the changes introduced to the basic model for the description of OP7/STV co-infection, related to Figure 2. (A) Basic model of OP7/STV co-infection that considers S7-OP7 as an additional viral genome segment, which encodes for M1-OP7. Furthermore, vRNPs bound by M1-STV and M1-OP7 are described separately. (B) Final model of OP7/STV co-infection that proposes multiple alterations to the infection kinetics to describe the observed accumulation of S7-OP7. These changes affect the transcription and replication of S7-OP7, the functionality of its associated protein M1-OP7, and the formation of progeny virions. Changes and extensions to the basic model are highlighted in red.

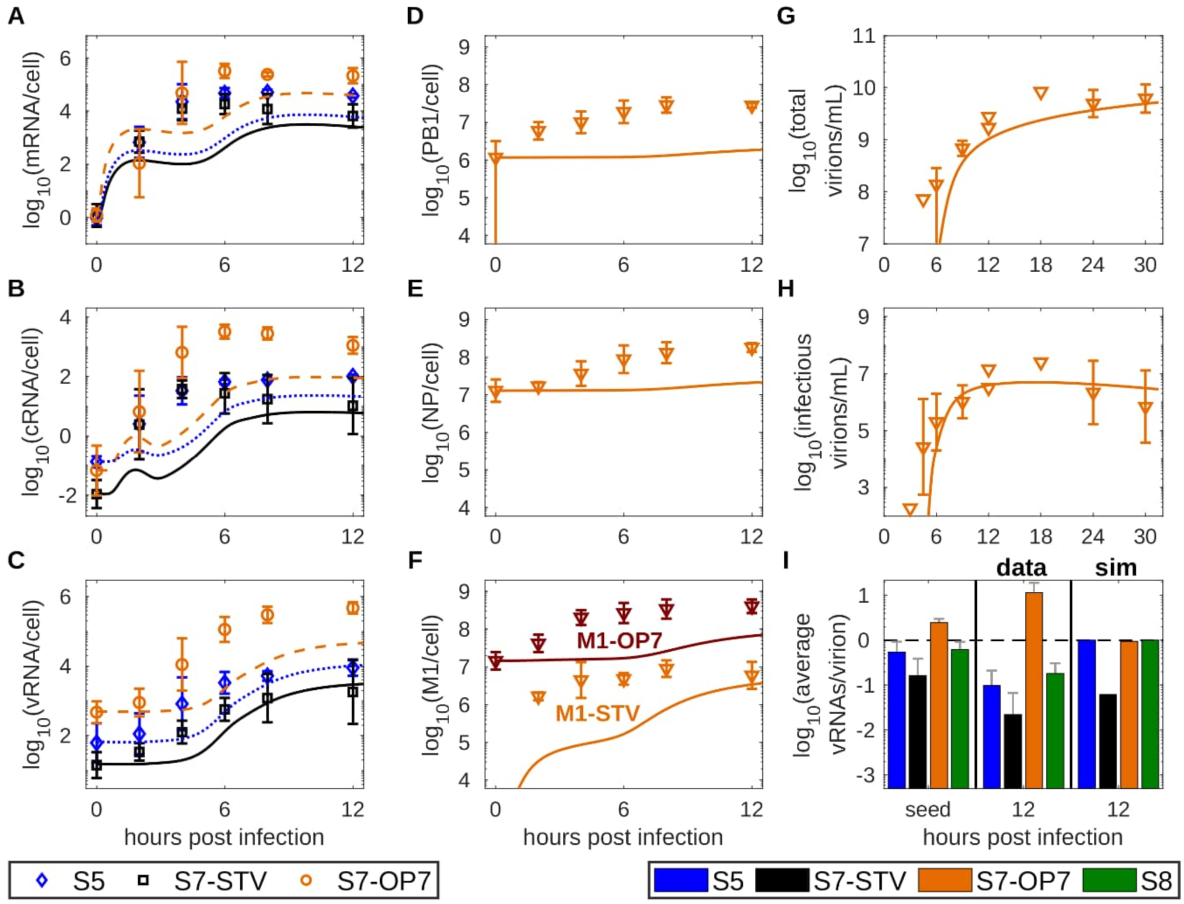

Figure S2. Simulation results of the initial OP7/STV co-infection model, related to Figure 1. Simulations of the initial OP7 model fitted to (A-C) cell-specific viral RNAs, (D-F) cell-specific viral proteins, (G-H) extracellular virus titers and (I) average vRNA levels in progeny virions measured in MDCK cells infected with an OP7 seed virus [S1]. Error bars represent the standard deviation of three independent experiments. Individual data points indicate data from a single or two independent experiments. (I) Normalized data (see STAR Methods) showing the average vRNA levels of virus particles for the seed virus and after infection compared to model simulations (sim).

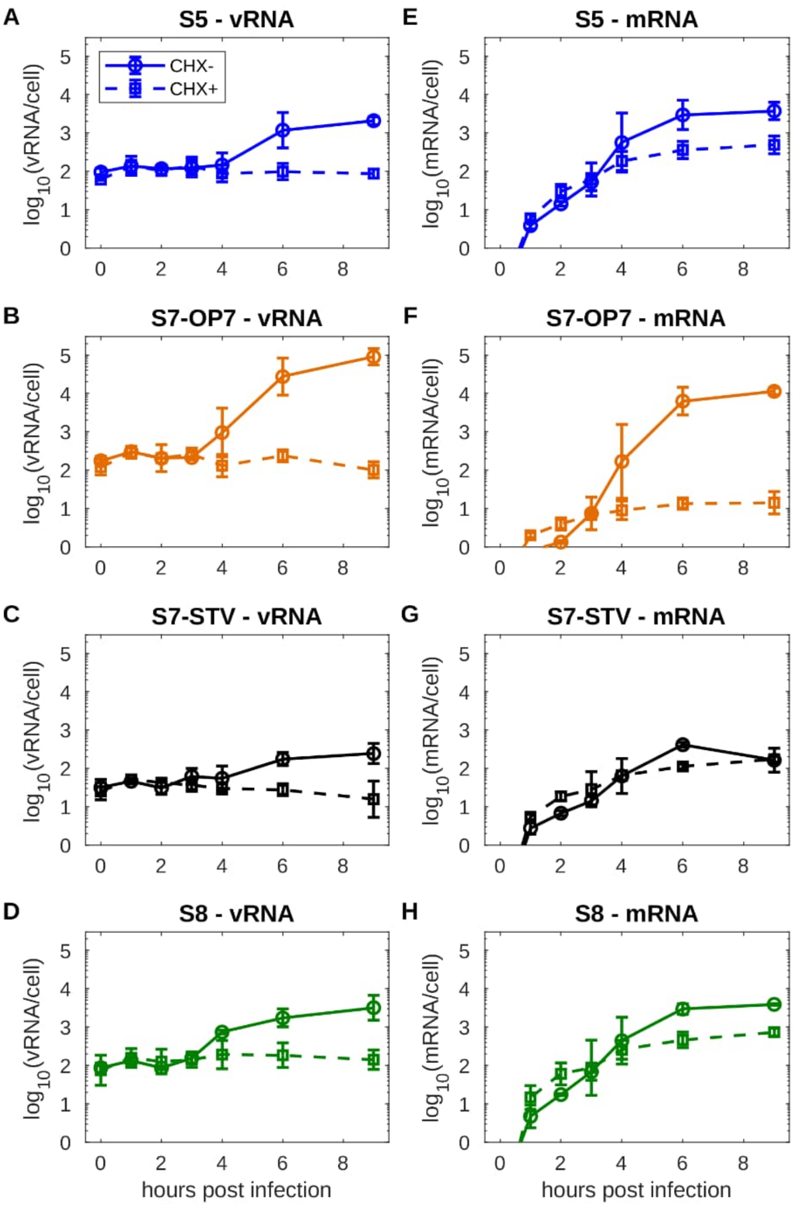

Figure S3. Accumulation of viral RNAs after *in vitro* OP7/STV co-infection with and without addition of cycloheximide, related to Figure 3. Time courses of (A-D) vRNA and (E-H) mRNA levels in MDCK cells infected by an OP7 seed virus with and without CHX for different viral genome segments. Error bars represent the standard deviation of two or three independent experiments.

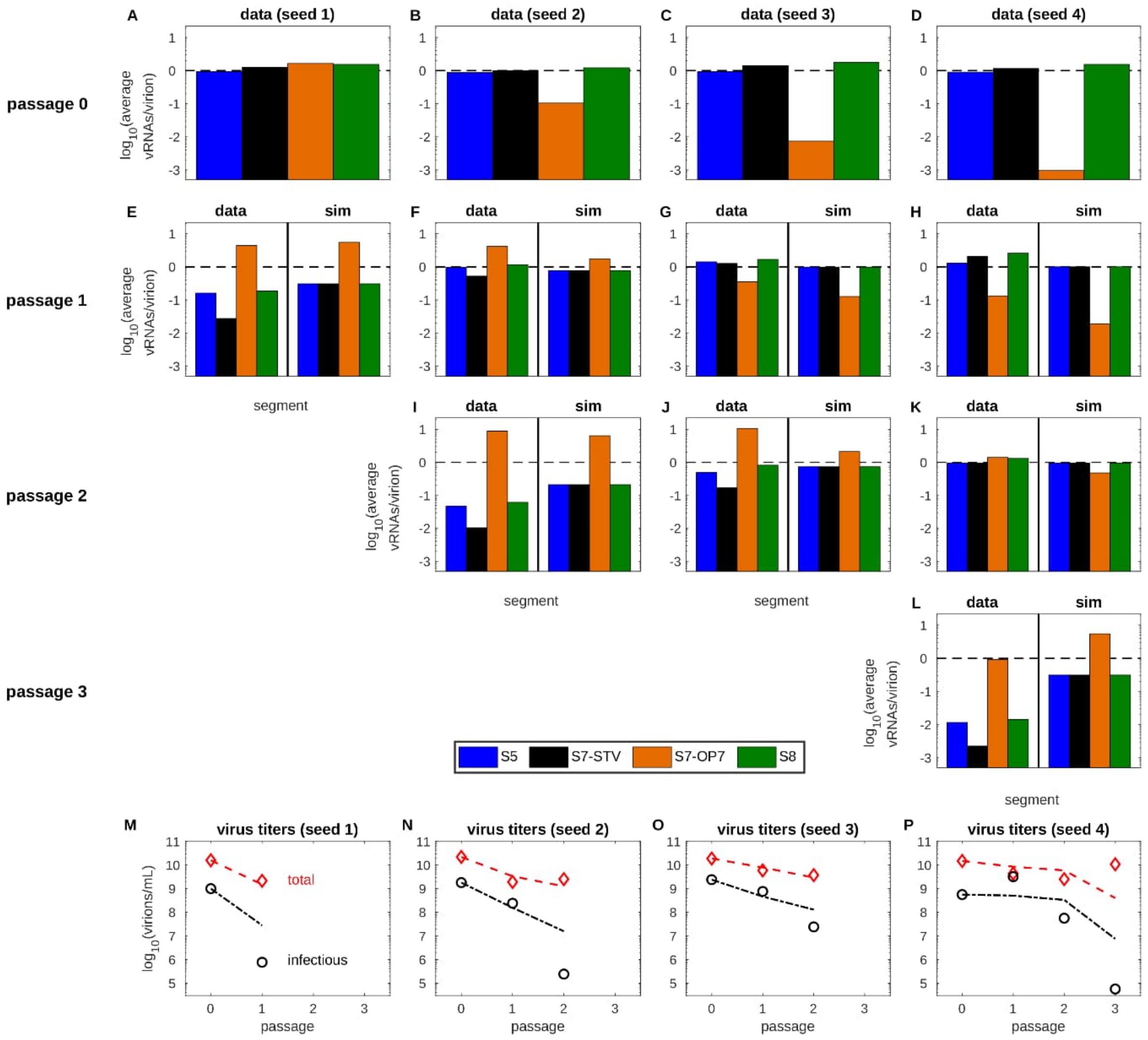

Figure S4. Model predictions of multiple *in vitro* co-infection passages considering a release of STV and OP7 particles, related to Figure 7. (A-D) Average vRNAs per virion of four segments for different OP7 seed viruses (passage 0). (E-L) Comparison of experimental data and predictions derived from an OP7/STV co-infection model that does not consider the release of “7+1” DIPs (sim). For simulation, the initial infection conditions were obtained from the original seed virus (passage 0) or the prediction of the previous virus passage. Measured vRNA levels were normalized (see STAR Methods). (M-P) Symbols represent total and infectious virus particle concentrations in the seed virus and after serial passaging. Curves depict model predictions.

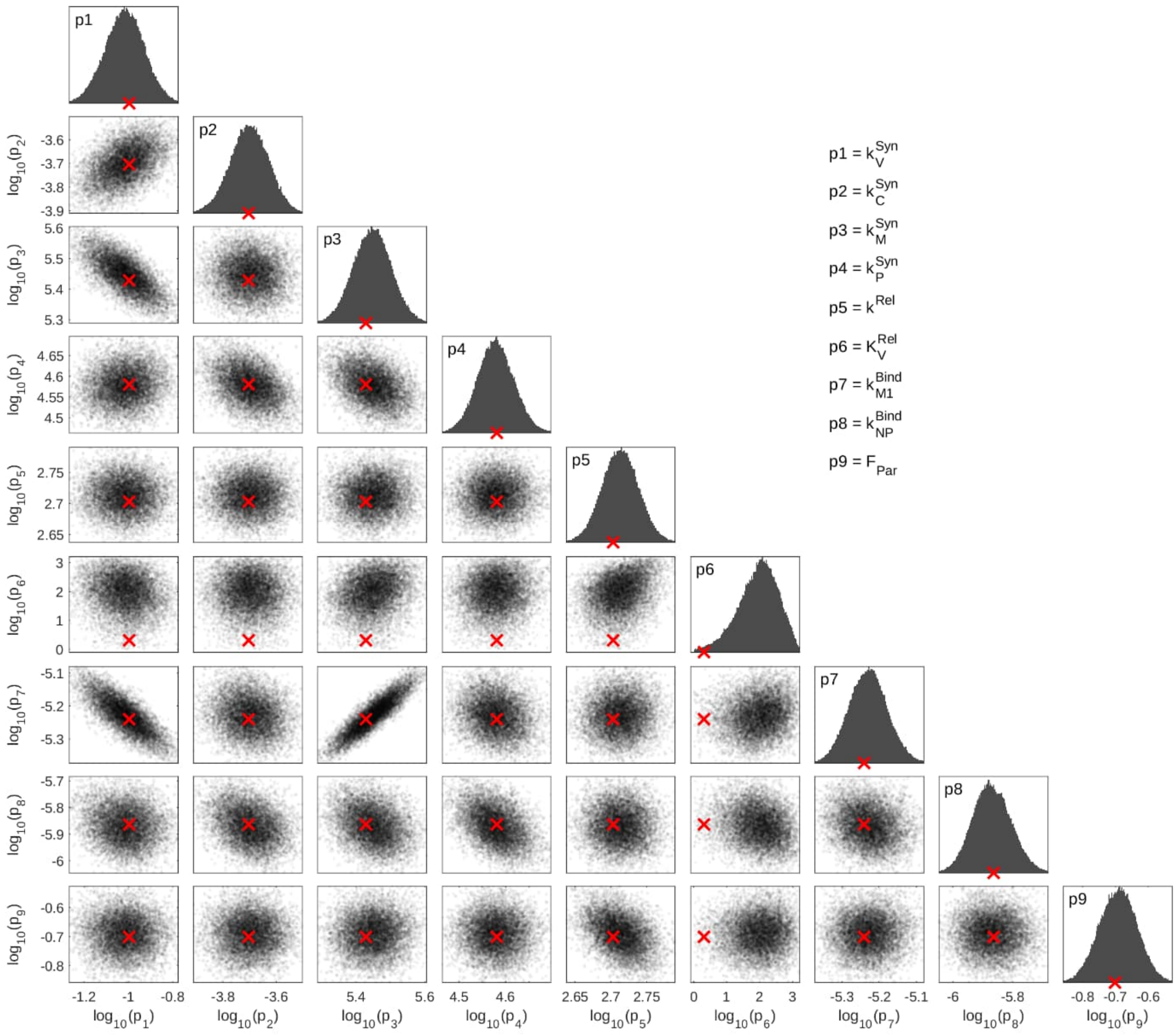

Figure S5. Parameter distributions and correlations for the calibration to STV infection data, related to STAR Methods. Histograms in the diagonal lane show the distributions of each individual estimated parameter. The scatter plots depict the dependency of two parameters. Posterior parameter distributions were determined using an MCMC approach (Bayesian inference). The red X-symbol represents the parameter value estimated using a global optimization algorithm (fSSm).

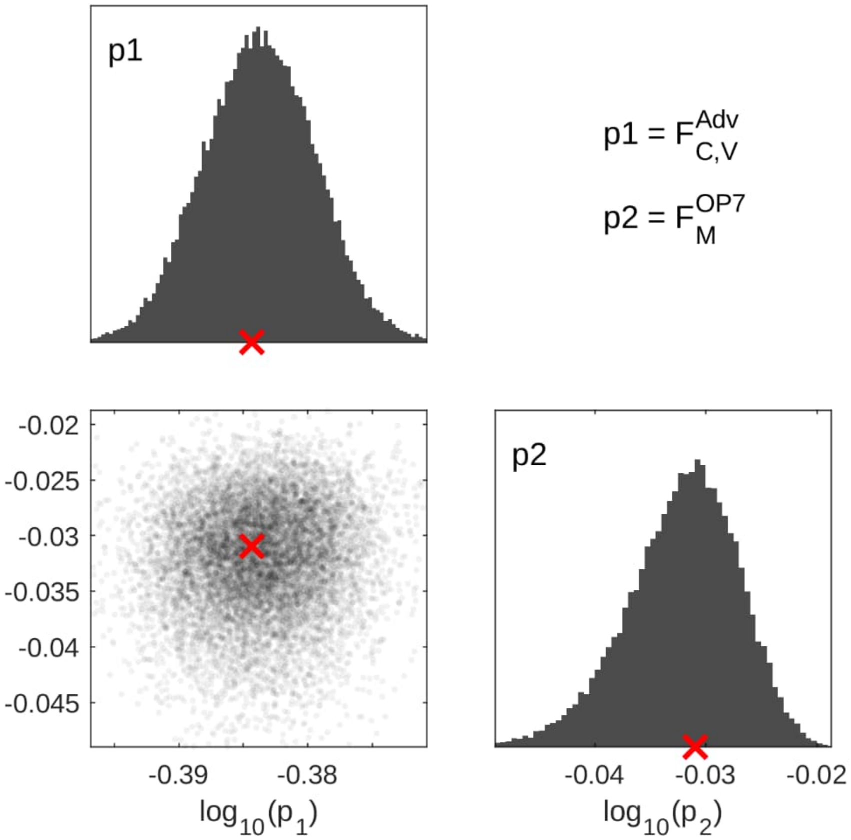

Figure S6. Parameter distributions and correlations for the calibration to OP7/STV co-infection data, related to STAR Methods. Histograms in the diagonal lane show the distributions of each individual parameter. The scatter plot depicts the dependency of the two parameters. Posterior parameter distributions were determined using an MCMC approach (Bayesian inference). The red X-symbol represents the parameter value estimated using a global optimization algorithm (fSSm).

Table S1. Parameters estimated from the experimental data, related to Figure 1.

Parameter		Value	Credible interval (95%)[a]
k_V^{Syn}	(h^{-1})	1.01×10^{-1}	$(0.64 – 1.44)\times10^{-1}$
k_C^{Syn}	(h^{-1})	1.98×10^{-4}	$(1.43 – 2.75)\times10^{-4}$
k_M^{Syn}	(nucleotides · h^{-1})	2.68×10^{5}	$(2.18 – 3.60)\times10^{5}$
k_P^{Syn}	(nucleotides · h^{-1})	3.81×10^{4}	$(3.17 – 4.55)\times10^{4}$
k^{Rel}	(virions · h^{-1})	5.05×10^{2}	$(4.61 – 5.81)\times10^{2}$
K_V^{Rel}	(virions)	2.03×10^{0}	$(1.00 – 1015.64)\times10^{0}$ [b]
k_{NP}^{Bind}	($molecules^{-1}$ · h^{-1})	1.37×10^{-6}	$(1.02 – 1.83)\times10^{-6}$
k_{M1}^{Bind}	($molecules^{-1}$ · h^{-1})	5.75×10^{-6}	$(4.70 – 7.47)\times10^{-6}$
F_{Par}	(–)	1.99×10^{-1}	$(1.56 – 2.64)\times10^{-1}$
$F_{C,V}^{Adv}$	(–)	4.13×10^{-1}	$(4.05 – 4.21)\times10^{-1}$
F_M^{OP7}	(–)	9.31×10^{-1}	$(9.06 – 9.47)\times10^{-1}$

[a] 95% credible intervals were determined from posterior parameter distributions calculated via Bayesian inference (Figures S5 and S6) using a Markov chain Monte Carlo sampler [S2].

[b] Estimates reached lower parameter bounds.

Table S2. Evaluation of the model fits performed for different hypotheses covered during model expansion, related to Figure 3.

Model version (replication)	Figure	No. of model parameters (fitted)	SSR	AIC	AICc
basic	S1	56 (9)	4236	516	643
M1 binding	3A-C	56 (9)	9944	609	737
RA + M1 binding	3E-G	57 (10)	814	336	470
RA + TR	5A-C	58 (11)	5176	542	683
RA + TR + M1 export	5D-F	58 (11)	5239	543	685
RA + TR + M1 packaging	5D-F	58 (11)	5176	542	683
M1 binding + RA + TR (final)	1, 3I-K, 4, 5G-I	58 (11)	482	280	422
**Model version (release)**	**Figure**	**No. of model parameters (fitted)**	**SSR**	**AIC**	**AICc**
STV + DIP	6C	58 (11)	46	128	1
STV + OP7 particle	6D	58 (11)	4	118	-8
STV + DIP + OP7 particle (final)	1, 6E	58 (11)	6	120	-7
**Model version (prediction)**	**Figure**	**No. of model parameters (fitted)**	**SSR**	**AIC**	**AICc**
STV + OP7 particle	S3	58 (11)	29	95	-495
STV + DIP + OP7 particle (final)	7	58 (11)	13	55	-535

SSR: sum of squared residuals (errors of each variable were normalized to the respective maximum measurement value);

AIC: Akaike information criterion

AICc: Akaike information criterion for small sample sizes

RA: replication advantage for S7-OP7 cRNA and vRNA

TR: transcription reduction for S7-OP7 mRNA

Table S3. Parameters of the OP7/STV co-infection model, related to STAR Methods.

Parameter	Description	Value	Unit	Source
B_{Hi}^{Tot}	number of high-affinity binding sites	150	sites	[S3]
B_{Lo}^{Tot}	number of low-affinity binding sites	1000	sites	[S3]
D_{Rib}	distance between two adjacent ribosomes	160	nucleotides	[S4]
F_{Fus}	fraction of fusion-competent virions	0.51	–	[S5]
F_{Par}	fraction of infectious STV particles	0.20	–	model fit in Fig 1 (see Table S1)
F_{Spl7}	fraction of M2-encoding mRNAs	2.00×10^{-2}	–	[S6]
F_{Spl8}	fraction of NEP-encoding mRNAs	0.125	–	[S7]
$F_{C,V}^{Adv}$	S7-OP7 cRNA and vRNA replication advantage factor	0.36	–	model fit in Fig 1 (see Table S1)
F_{M}^{OP7}	reduction factor of S7-OP7 mRNA synthesis	0.93	–	model fit in Fig 1 (see Table S1)
F_{M}^{RdRp}	reduction factor for RdRp-related viral mRNA synthesis	0.12	–	[S8]
$K_{V^{Rel}}$	influence of available viral components on virion release	2.03	virions	model fit in Fig 1 (see Table S1)
k_{Hi}^{Att}	attachment to high-affinity binding sites	8.09×10^{-2}	$site^{-1}\cdot h^{-1}$	[S6]
k_{Lo}^{Att}	attachment to low-affinity binding sites	4.55×10^{-4}	$site^{-1}\cdot h^{-1}$	[S6]
k_{M1}^{Bind}	binding of M1-STV to nuclear vRNPs	1.37×10^{-6}	$molecules^{-1}\cdot h^{-1}$	model fit in Fig 1 (see Table S1)
k_{NP}^{Bind}	binding of NP to RdRp-RNA complexes	5.75×10^{-6}	$molecules^{-1}\cdot h^{-1}$	model fit in Fig 1 (see Table S1)
k^{Cplx}	formation rate of vRNP complexes	1	$molecules^{-7}\cdot h^{-1}$	[S9]
k_{M}^{Deg}	degradation of mRNA	0.33	h^{-1}	[S5]
k_{Rnp}^{Deg}	degradation of RNPs	9.00×10^{-2}	h^{-1}	[S5]
k_{RRdRp}^{Deg}	degradation of RdRp-RNA complexes	4.25	h^{-1}	[S5]
k^{En}	endocytosis	4.8	h^{-1}	[S5]
k_{Hi}^{Eq}	equilibrium constant of high-affinity sites	1.13×10^{-2}	$sites^{-1}$	[S3]

Parameter	Description	Value	Unit	Source
$k_{\mathrm{Lo}}^{\mathrm{Eq}}$	equilibrium constant of low-affinity sites	8.33×10^{-5}	sites^{-1}	[S3]
k^{Fus}	fusion with endosomes	3.21	h^{-1}	[S5]
k^{Imp}	nuclear import	6	h^{-1}	[S10]
k^{RdRp}	formation of RdRp-complexes	1	$\mathrm{molecules}^{-2}\cdot\mathrm{h}^{-1}$	[S6]
k^{Rel}	virion release/budding	505	$\mathrm{virions}\cdot\mathrm{h}^{-1}$	model fit in Fig 1 (see Table S1)
$k_{\mathrm{C}}^{\mathrm{Syn}}$	cRNA synthesis	1.98×10^{-4}	h^{-1}	model fit in Fig 1 (see Table S1)
$k_{\mathrm{M}}^{\mathrm{Syn}}$	mRNA synthesis	2.68×10^{5}	$\mathrm{nucleotides}\cdot\mathrm{h}^{-1}$	model fit in Fig 1 (see Table S1)
$k_{\mathrm{P}}^{\mathrm{Syn}}$	protein synthesis	3.81×10^{4}	$\mathrm{nucleotides}\cdot\mathrm{h}^{-1}$	model fit in Fig 1 (see Table S1)
$k_{\mathrm{V}}^{\mathrm{Syn}}$	vRNA synthesis	0.10	h^{-1}	model fit in Fig 1 (see Table S1)
L_1	length of segment 1's mRNA	2320	nucleotides	[S11]
L_2	length of segment 2's mRNA	2320	nucleotides	[S11]
L_3	length of segment 3's mRNA	2211	nucleotides	[S11]
L_4	length of segment 4's mRNA	1757	nucleotides	[S11]
L_5	length of segment 5's mRNA	1540	nucleotides	[S11]
L_6	length of segment 6's mRNA	1392	nucleotides	[S11]
L_7	length of segment 7's unspliced mRNA	1005	nucleotides	[S11]
L_8	length of segment 8's unspliced mRNA	868	nucleotides	[S11]
L_9	length of OP7 segment 7's unspliced mRNA	1005	nucleotides	same length as STV segment 7
$L_{\mathrm{V},1}$	length of segment 1's vRNA and cRNA	2341	nucleotides	[S11]
$L_{\mathrm{V},2}$	length of segment 2's vRNA and cRNA	2341	nucleotides	[S11]
$L_{\mathrm{V},3}$	length of segment 3's vRNA and cRNA	2233	nucleotides	[S11]
$L_{\mathrm{V},4}$	length of segment 4's vRNA and cRNA	1778	nucleotides	[S11]
$L_{\mathrm{V},5}$	length of segment 5's vRNA and cRNA	1565	nucleotides	[S11]
$L_{\mathrm{V},6}$	length of segment 6's vRNA and cRNA	1413	nucleotides	[S11]
$L_{\mathrm{V},7}$	length of segment 7's vRNA and cRNA	1027	nucleotides	[S11]

Parameter	Description	Value	Unit	Source
$L_{V,8}$	length of segment 8’s vRNA and cRNA	890	nucleotides	[S11]
$L_{V,9}$	length of OP7 segment 7 vRNA and cRNA	1027	nucleotides	same length as STV segment 7
$N_{P_{HA}}$	number of HA molecules in a virion	233	molecules·virion^{-1}	[S12]
$N_{P_{NA}}$	number of NA molecules in a virion	30	molecules·virion^{-1}	[S12]
$N_{P_{M1}}$	number of M1 molecules in a virion	2700	molecules·virion^{-1}	[S12]
$N_{P_{M2}}$	number of M2 molecules in a virion	3	molecules·virion^{-1}	[S12]
N_{STV}^{In}	average STV segment vRNA in infecting virions	0.58	–	calculated based on S1 Data
$N_{S7\text{-}STV}^{In}$	average S7-STV segment vRNA in infecting virions	0.16	–	calculated based on S1 Data
$N_{S7\text{-}OP7}^{In}$	average S7-OP7 segment vRNA in infecting virions	2.46	–	calculated based on S1 Data
N_{M1}^{Nuc}	nucleotides bound by one M1 molecule	200	nucleotides	[S13]
N_{NP}^{Nuc}	nucleotides bound by one NP molecule	24	nucleotides	[S14]
N_{OP7}^{Par}	number of S7-OP7 vRNPs in OP7 particle	8	molecules	analogous to STV particles

Table S4: Tagged primers for RT, related to STAR Methods.

Target	RNA type	Primer name	Sequence (5’ -> 3’)
S5	vRNA	S5 tagRT for	ATTTAGGTGACACTATAGAAGCGAGTGATTA TGAGGGACGGTTGAT
	cRNA	S5 tagRT rev	GCTAGCTTCAGCTAGGCATCAGTAGAAACA AGGGTATTTTTCTT
S7-STV	vRNA	S7 wt3 tagRT for	ATTTAGGTGACACTATAGAAGCGTCTCGCT ATTGCCGCAAA
	cRNA	S7 wt tagRT rev	GCTAGCTTCAGCTAGGCATCAGTAGAAACA AGGTAGTTTTTTAC
S7-OP7	vRNA	S7-OP7 tagRT for	ATTTAGGTGACACTATAGAAGCGACTGTGA CTGCTGAAGTGGTG
	cRNA	S7-OP7 tagRT rev	GCTAGCTTCAGCTAGGCATCAGTAGAAACA AGGTAGTTTTTTACTC
S8	vRNA	S8 tagRT for	ATTTAGGTGACACTATAGAAGCGGATAGTG GAGCGGATTCTG
	cRNA	S8 tagRT rev	GCTAGCTTCAGCTAGGCATC AGTAGAAACAAGGGTGTTTTTTAG
S5, S7-STV, S7-OP7, S8	mRNA	Oligo tagdTRT	GTAAAACGACGGCCAGTTTTTTTTTTTTTTT TT

Table S5: Primers for real-time qPCR, related to STAR Methods.

Target	RNA type	Primer name	Sequence (5’ -> 3’)
Introduced tag sequence	vRNA	vRNA tagRealtime for	ATTTAGGTGACACTATAGAAGCG
	cRNA	cRNA tagRealtime rev	GCTAGCTTCAGCTAGGCATC
	mRNA	mRNA tagRealtime rev	GTAAAACGACGGCCAGT
S5	vRNA	S5 Realtime rev	CGCACTGGGATGTTCTTC
	cRNA and mRNA	S5 Realtime for	GGAAAGTGCAAGACCAGAAGAT
S7-STV	vRNA	S7 wt3 realtime rev	CCTTTCAGTCCGTATTTAAAGC
	cRNA and mRNA	S7 wt realtime for 1	CGCTTTAAATACGGACTGAAAG
S7-OP7	vRNA	S7-OP7 Realtime rev	CATTTGCCTAGCCCGAATC
	cRNA and mRNA	S7-OP7 Realtime for	CCTCTCGTTATTGCCTCAAG
S8	vRNA	S8 Realtime rev	CACTTTCTGCTTGGGTATGA
	cRNA and mRNA	S8 Realtime for	GGCGGGAACAATTAGGTCAGA

Table S6: Primers for RNA reference standard generation, related to STAR Methods.

Target	RNA type	Primer name	Sequence (5' -> 3')
S5	cRNA	S5 Uni T7 for	TAATACGACTCACTATAGGGAGCAAAAGCAGGGTAGATAATC
		S5 Uni rev	AGTAGAAACAAGGGTATTTTTC
	vRNA	S5 Uni for	AGCAAAAGCAGGGTAGATAATC
		S5 Uni T7 rev	TAATACGACTCACTATAGGGAGTAGAAACAAGGGTATTTTTC
	mRNA	S5 Uni T7 for	TAATACGACTCACTATAGGGAGCAAAAGCAGGGTAGATAATC
		S5 dT rev	TTTTTTTTTTTTTTTTCTTTAATTGTC
S7-STV	cRNA	S7 Uni T7 for neu	TAATACGACTCACTATAGGGAAGCGAAAGCAGGTAGATATTG
		S7 Uni rev neu	AGTAGAAACAAGGTAGTTTTTTAC
	vRNA	S7 Uni for neu	AGCGAAAGCAGGTAGATATTG
		S7 Uni T7 rev neu	TAATACGACTCACTATAGGGAAGTAGAAACAAGGTAGTTTTTTAC
	mRNA	S7 Uni T7 for neu	TAATACGACTCACTATAGGGAAGCGAAAGCAGGTAGATATTG
		S7 dT rev	TTTTTTTTTTTTTTTTACTCCAGCTCT
S7-OP7	cRNA	S7-OP7 Uni T7 for	TAATACGACTCACTATAGGGAGTAAAAACAGGTAGATGTTGAAAG
		S7-OP7 Uni rev	AGTAGAAACAAGGTAGTTTTTTAC
	vRNA	S7-OP7 Uni for	AGTAAAAACAGGTAGATGTTGAAAG
		S7-OP7 Uni T7 rev	TAATACGACTCACTATAGGGAGTAGAAACAAGGTAGTTTTTTAC
	mRNA	S7-OP7 Uni T7 for	TAATACGACTCACTATAGGGAGTAAAAACAGGTAGATGTTGAAAG
		S7-OP7 dT rev	TTTTTTTTTTTTTTTTACTCCAGCTCTATGC
S8	cRNA	S8 Uni T7 for	TAATACGACTCACTATAGGGAGAAAAAGCAGGGTGACAAA
		S8 Uni rev	AGTAGAAACAAGGGTGTTTT
	vRNA	S8 Uni for	AGAAAAAGCAGGGTGACAAA
		S8 Uni T7 rev	TAATACGACTCACTATAGGGAAGTAGAAACAAGGGTGTTTT
	mRNA	S8 Uni T7 for	TAATACGACTCACTATAGGGAGAAAAAGCAGGGTGACAAA
		S8 dT rev	TTTTTTTTTTTTTTTTAGTACTAAATAAGCTGAAACGAG

Supplementary references

S1. Kupke, S.Y., Riedel, D., Frensing, T., Zmora, P., and Reichl, U. (2019). A Novel Type of Influenza A Virus-Derived Defective Interfering Particle with Nucleotide Substitutions in Its Genome. J Virol *93*. 10.1128/jvi.01786-18.

S2. Goodman, J., and Weare, J. (2010). Ensemble samplers with affine invariance. Communications in Applied Mathematics and Computational Science *5*, 65-80. 10.2140/camcos.2010.5.65.

S3. Nunes-Correia, I., Ramalho-Santos, J., Nir, S., and Pedroso de Lima, M.C. (1999). Interactions of influenza virus with cultured cells: detailed kinetic modeling of binding and endocytosis. Biochemistry *38*, 1095-1101. 10.1021/bi9812524.

S4. Arava, Y., Wang, Y., Storey, J.D., Liu, C.L., Brown, P.O., and Herschlag, D. (2003). Genome-wide analysis of mRNA translation profiles in Saccharomyces cerevisiae. Proc Natl Acad Sci U S A *100*, 3889-3894. 10.1073/pnas.0635171100.

S5. Heldt, F.S., Frensing, T., and Reichl, U. (2012). Modeling the intracellular dynamics of influenza virus replication to understand the control of viral RNA synthesis. J Virol *86*, 7806-7817. 10.1128/jvi.00080-12.

S6. Heldt, F.S., Frensing, T., Pflugmacher, A., Gröpler, R., Peschel, B., and Reichl, U. (2013). Multiscale modeling of influenza A virus infection supports the development of direct-acting antivirals. PLoS Comput Biol *9*, e1003372. 10.1371/journal.pcbi.1003372.

S7. Robb, N.C., Jackson, D., Vreede, F.T., and Fodor, E. (2010). Splicing of influenza A virus NS1 mRNA is independent of the viral NS1 protein. J Gen Virol *91*, 2331-2340. 10.1099/vir.0.022004-0.

S8. Rüdiger, D., Kupke, S.Y., Laske, T., Zmora, P., and Reichl, U. (2019). Multiscale modeling of influenza A virus replication in cell cultures predicts infection dynamics for highly different infection conditions. PLoS Comput Biol *15*, e1006819. 10.1371/journal.pcbi.1006819.

S9. Laske, T., Heldt, F.S., Hoffmann, H., Frensing, T., and Reichl, U. (2016). Modeling the intracellular replication of influenza A virus in the presence of defective interfering RNAs. Virus Res *213*, 90-99. 10.1016/j.virusres.2015.11.016.

S10. Babcock, H.P., Chen, C., and Zhuang, X. (2004). Using single-particle tracking to study nuclear trafficking of viral genes. Biophys J *87*, 2749-2758. 10.1529/biophysj.104.042234.

S11. Lamb, R.A., and Krug, R.M. (2001). Orthomyxoviridae: the viruses and their replication. In Fields Virology, D.M. Knipe, and P.M. Howley, eds. (Lippincott Williams & Wilkins), pp. 1487-1531.

S12. Hutchinson, E.C., Charles, P.D., Hester, S.S., Thomas, B., Trudgian, D., Martínez-Alonso, M., and Fodor, E. (2014). Conserved and host-specific features of influenza virion architecture. Nat Commun *5*, 4816. 10.1038/ncomms5816.

S13. Wakefield, L., and Brownlee, G.G. (1989). RNA-binding properties of influenza A virus matrix protein M1. Nucleic Acids Res *17*, 8569-8580. 10.1093/nar/17.21.8569.

S14. Portela, A., and Digard, P. (2002). The influenza virus nucleoprotein: a multifunctional RNA-binding protein pivotal to virus replication. J Gen Virol *83*, 723-734. 10.1099/0022-1317-83-4-723.
